# Loss of AID exacerbates the malignant progression of CLL

**DOI:** 10.1038/s41375-022-01663-5

**Published:** 2022-08-30

**Authors:** Avery C. Lee, Sai Ravi Pingali, Javier A. Pinilla-Ibarz, Michael L. Atchison, Constantinos Koumenis, Yair Argon, Andrei Thomas-Tikhonenko, Carl De Trez, Chih-Chi Andrew Hu, Chih-Hang Anthony Tang

**Affiliations:** 1 Center for Translational Research in Hematologic Malignancies, Houston Methodist Cancer Center, Houston Methodist Research Institute, Houston, TX USA; 2 Cell & Molecular Biology Graduate Group, Perelman School of Medicine, University of Pennsylvania, Philadelphia, PA USA; 3 Department of Malignant Hematology, H. Lee Moffitt Cancer Center & Research Institute, Tampa, FL USA; 4 Department of Biomedical Sciences, School of Veterinary Medicine, University of Pennsylvania, Philadelphia, PA USA; 5 Department of Radiation Oncology, Perelman School of Medicine, University of Pennsylvania, Philadelphia, PA USA; 6 Department of Pathology and Laboratory Medicine, Perelman School of Medicine, University of Pennsylvania, Philadelphia, PA USA; 7 Division of Cell Pathology, Children’s Hospital of Philadelphia, Philadelphia, PA USA; 8 Division of Cancer Pathobiology, Children’s Hospital of Philadelphia, Philadelphia, PA USA; 9 Laboratory of Cellular and Molecular Immunology, Vrije Universiteit Brussel, Brussels, Belgium

**Keywords:** Chronic lymphocytic leukaemia, Cancer models

## Abstract

Activation-induced cytidine deaminase (AID) has been implicated as both a positive and a negative factor in the progression of B cell chronic lymphocytic leukemia (CLL), but the role that it plays in the development and progression of this disease is still unclear. We generated an AID knockout CLL mouse model, AID^−/−^/Eμ-TCL1, and found that these mice die significantly earlier than their AID-proficient counterparts. AID-deficient CLL cells exhibit a higher ER stress response compared to Eμ-TCL1 controls, particularly through activation of the IRE1/XBP1s pathway. The increased production of secretory IgM in AID-deficient CLL cells contributes to their elevated expression levels of XBP1s, while secretory IgM-deficient CLL cells express less XBP1s. This increase in XBP1s in turn leads AID-deficient CLL cells to exhibit higher levels of B cell receptor signaling, supporting leukemic growth and survival. Further, AID^−/−^/Eμ-TCL1 CLL cells downregulate the tumor suppressive SMAD1/S1PR2 pathway and have altered homing to non-lymphoid organs. Notably, CLL cells from patients with IgHV-unmutated disease express higher levels of XBP1s mRNA compared to those from patients with IgHV-mutated CLL. Our studies thus reveal novel mechanisms by which the loss of AID leads to worsened CLL and may explain why unmutated CLL is more aggressive than mutated CLL.

## Introduction

One of the major prognostic indicators for chronic lymphocytic leukemia (CLL) is the mutational status of the immunoglobulin heavy chain locus (IgHV). Patients with mutated IgHV have longer survival and a better response to chemotherapy than those with unmutated IgHV [1, 2]. This immunoglobulin mutation status is mediated by activation-induced cytidine deaminase (AID) [3], an enzyme primarily expressed in B cells and during the germinal reaction when two gene-modifying processes critical for antibody function occur: class switch recombination and somatic hypermutation [4, 5]. AID activity allows B cells to develop a diverse repertoire of antibodies to combat pathogens, but in the context of CLL, AID may have conflicting roles [6]. In human patients, it has been shown that there is a correlation between higher levels of AID mRNA and a worse prognosis, although circulating CLL cells do not express AID protein [7–9]. In contrast, the expression of AID has been shown to be targetable to kill CLL cells [10] and may be important in the development of the relatively indolent mutated CLL [11–14]. The function of AID in the progression of mutated versus unmutated CLL is not well understood, and the molecular mechanisms behind the development of CLL in an AID-deficient background have not been thoroughly studied.

As antibody-secreting cells, B cells depend on the endoplasmic reticulum (ER) and the ER stress response to correctly produce and fold immunoglobulins [15]. Of primary importance to B cells is the ER stress sensing pathway composed of inositol-requiring enzyme 1 (IRE1) and X-box binding protein 1 (XBP1) [16–18]. IRE1 is an ER-resident transmembrane protein which, when activated by ER stress, has endonuclease activity that can excise 26 nucleotides from the mRNA of XBP1, resulting in a spliced XBP1 mRNA encoding a transcription factor XBP1s [19–21]. After translocating to the nucleus, XBP1s upregulates the transcription of chaperones and lipids to expand the ER protein folding capacity and clear the stress [22]. We have previously shown that the endoplasmic reticulum stress response supports leukemic progression in CLL [23], and the most conserved IRE1/XBP1s pathway is critically important for the malignant progression of CLL [24]. In B cells, XBP1s expression leads to the production of lipids such as sphingomyelin and phosphatidylinositol, promoting B cell receptor (BCR) signaling [17, 18] which supports CLL growth [24].

## Results

### AID^−/−^/MD4^+/−^/Eμ-TCL1 mice fail to recognize HEL and die significantly earlier than their AID-proficient counterparts

Previously, to investigate the role of a monoclonal BCR in the progression of CLL in mice, we generated MD4^+/−^/Eμ-TCL1 mice by crossing Eμ-TCL1 mice, which spontaneously develop CLL with all the clinical features of aggressive human CLL [25, 26], with MD4^+/−^ transgenic mice, which produce a monoclonal BCR against HEL [27–29]. However, we found that although B220+/CD5− B cells were able to bind to HEL, the B220^low^/CD5+ CLL cells in older MD4^+/−^/Eμ-TCL1 mice failed to recognize HEL. As AID is an immunoglobulin mutating enzyme, we hypothesized that aberrant expression of AID in CLL cells could be causing mutations in the MD4 transgene, leading to the loss of HEL recognition. To determine if this was the case, we crossed the MD4^+/−^/Eμ-TCL1 mice with AID^−/−^ mice [4]. As in the MD4^+/−^/Eμ-TCL1 mice, B cells were able to recognize HEL, but CLL cells in older AID^−/−^/MD4^+/−^/Eμ-TCL1 mice failed to bind to HEL, indicating that AID was not causing the loss of HEL recognition (Fig. S1A). Strikingly, these AID-deficient MD4^+/−^/Eμ-TCL1 mice died significantly earlier than their AID-proficient counterparts, with a median survival of 208 versus 248 days (Fig. S1B). This survival difference was seen in both sexes (Fig. S1C, D). When stimulated with the Toll-like receptor (TLR) 4 ligand, lipopolysaccharide (LPS), B cells from MD4^+/−^/Eμ-TCL1 but not AID^−/−^/MD4^+/−^/Eμ-TCL1 mice could express AID protein (Fig. S1E).

### AID-deficient Eμ-TCL1 mice die at a younger age than AID-proficient Eμ-TCL1 mice, but do not exhibit an increased CLL burden in the blood

**Fig. 1:**
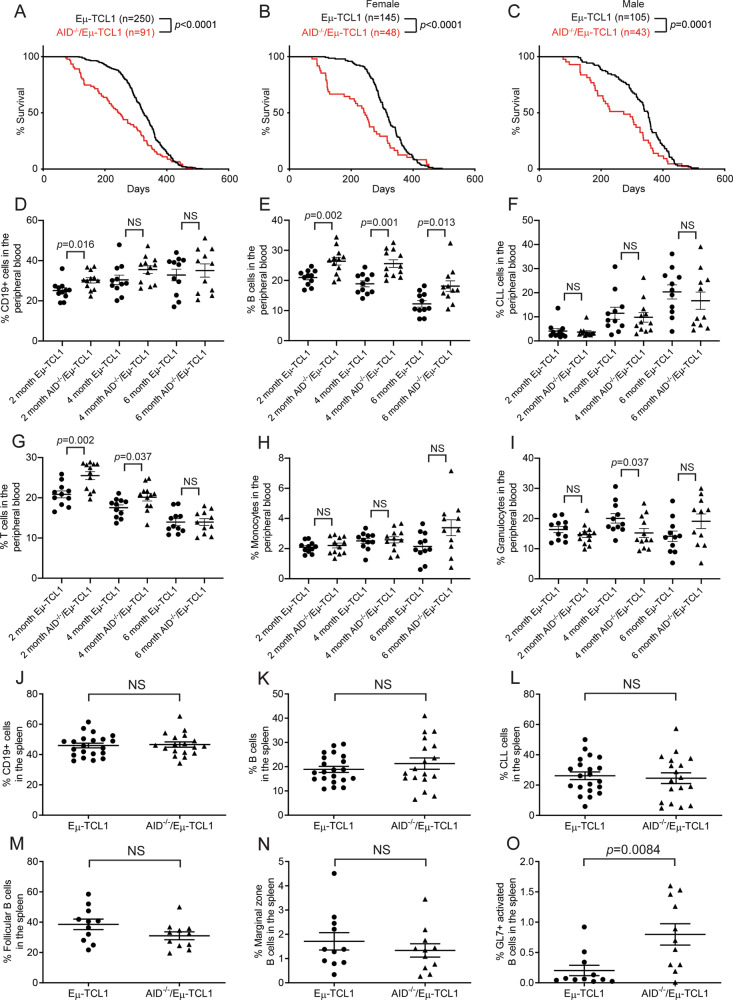
AID^−/−^/Eμ-TCL1 mice died significantly earlier than Eμ-TCL1 mice and had increased GL7+ activated B cells in the spleens. **A** Kaplan-Meier survival analysis of Eμ-TCL1 and AID^−/−^/Eμ-TCL1 mice. **B**, **C** Kaplan–Meier survival analysis of female (**B**) and male (**C**) Eμ-TCL1 and AID^−/−^/Eμ-TCL1 mice. **D**–**I** Quantification of CD19+ B cell lineage cells (**D**), B cells (**E**), CLL cells (**F**), T cells (**G**), monocytes (**H**), and granulocytes (**I**) in the peripheral blood of Eμ-TCL1 (*n* = 11) and AID^−/−^/Eμ-TCL1 (*n* = 12 at 2 and 4 months and *n* = 11 at 6 months) mice at 2, 4, and 6 months of age by flow cytometry. **J**–**L** Quantification of CD19+ B cell lineage cells (**J**), B cells (**K**), and CLL cells (**L**) in the spleens of 6-month-old Eμ-TCL1 (*n* = 21) and AID^−/−^/Eμ-TCL1 (*n* = 18) mice by flow cytometry. **M**–**O** Quantification of follicular B cells (**M**), marginal zone B cells (**N**), and GL7+ activated B cells (**O**) in the spleens of 6-month-old Eμ-TCL1 (*n* = 11) and AID^−/−^/Eμ-TCL1 (*n* = 11) mice by flow cytometry.

To investigate if AID deficiency contributed to an earlier death from CLL without the complication of the MD4 transgene, we generated AID^−/−^/Eμ-TCL1 mice. Indeed, the AID^−/−^/Eμ-TCL1 mice died significantly earlier than the Eμ-TCL1 controls with a median survival of 245 versus 324 days (Fig. 1A). Again, this survival difference was seen in both sexes (Fig. 1B, C). As in Eμ-TCL1 mice, AID^−/−^/Eμ-TCL1 mice accumulated a population of CD19+/B220^low^/CD5+ CLL cells in the spleens during leukemic progression, eventually making up a majority of the splenocytes (Fig. S2A). Additionally, these mice were functionally AID-deficient, as they had increased secretory IgM in the serum but were unable to produce class-switched secretory IgG or IgA (Fig. S2B–D). To examine the impact of AID deficiency on immune cell compartments during the progression of CLL, we followed the CD19+ populations in the peripheral blood by flow cytometry as mice aged. AID^−/−^/Eμ-TCL1 mice had higher CD19+/B220+/CD5− B cell populations at 2, 4, and 6 months of age (Fig. 1D, E). There was not a statistically significant difference in CD19+/B220^low^/CD5+ CLL cells between Eμ-TCL1 and AID^−/−^/Eμ-TCL1 mice (Fig. 1D, F). AID^−/−^/Eμ-TCL1 mice had a significant increase in the percentage of CD3+ T cells at two and four months of age (Fig. 1G). AID^−/−^/Eμ-TCL1 mice did not have a statistically significant difference in percentages of monocytes and granulocytes except for a decrease in granulocytes at four months of age (Fig. 1H, I). Using a complete blood count to enumerate absolute numbers of blood cells, we found that AID^−/−^/Eμ-TCL1 mice had similar numbers of lymphocytes as Eμ-TCL1 mice, but had significantly lower numbers of monocytes and granulocytes (Fig. S3A–C), suggesting that the increased T cells may be attributed to the decreased myeloid populations.

### AID^−/−^/Eμ-TCL1 mice have higher percentages of GL7+ activated B cells and CD4+ T cells in the spleens

We next examined the percentages of different immune cells in the spleens of 6-month-old Eμ-TCL1 and AID^−/−^/Eμ-TCL1 mice. There was not a statistically significant difference in percentages of the CD19+ B cell lineage, B220+/CD5− B cells, or B220^low^/CD5+ CLL cells (Figs. 1J–L, S4A). We next looked at the gated CD19+ B cell subsets and detected no statistically significant difference between these mice in the GL7-/AA4.1-/CD1d-/CD23+ follicular B cells and GL7-/AA4.1-/CD1d+/CD23− marginal zone B cells (Figs. 1M, N, S4B). AID^−/−^/Eμ-TCL1 mice produced more GL7+ activated germinal center (GC) B cells than Eμ-TCL1, which is a characteristic of AID deficiency (Fig. 1O) [4]. We next examined the T cell compartments and observed that the percentages of CD3+ total T cells and CD3+/CD4+ helper T cells but not CD3+/CD8+ cytotoxic T cells were significantly increased in the AID^−/−^/Eμ-TCL1 mice (Fig. S5A–D). The CD4+ T cells were examined for FOXP3+/CD25+ regulatory T cells but there was not a significant difference in percentages (Fig. S5E, F). CD4+ T cells have been shown to promote the survival and proliferation of CLL cells in the Eμ-TCL1 mouse model [30]. We also investigated myeloid cells in the spleens and found no significant differences in percentages of CD11b+/Ly6G+ granulocytes, CD11b+/Ly6C+ monocytes, and CD11c+ dendritic cells (Fig. S6A–D). Previously, we found that secretory IgM causes the recruitment of myeloid-derived suppressor cells (MDSCs) to the tumor microenvironment, exacerbating CLL progression [28]. AID^−/−^/Eμ-TCL1 mice have significantly higher levels of serum IgM, but such levels are not sufficient in inducing further accumulation of MDSCs and Ly6G+ MDSCs isolated from the spleens of these mice were not significantly more suppressive of T cell proliferation than those from Eμ-TCL1 mice (Fig. S6E, F).

### AID^−/−^/Eμ-TCL1 CLL cells have lower expression of B220 and higher BCR signaling

**Fig. 2:**
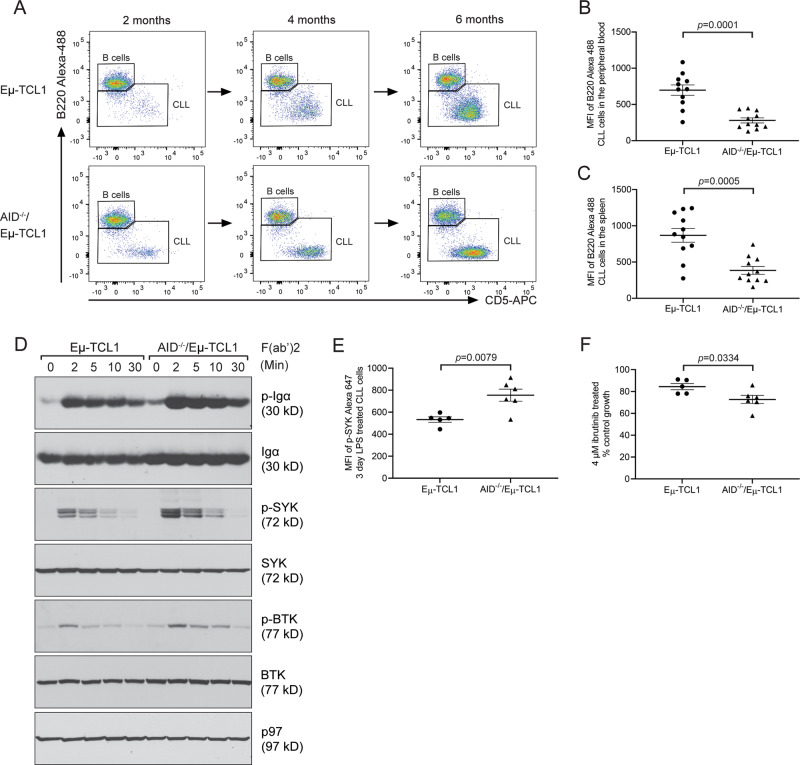
AID-deficient CLL cells have lower B220 and higher BCR signaling. **A** Representative flow cytometry plots of B220 expression in B cells and CLL cells from the peripheral blood of Eμ-TCL1 and AID^−/−^/Eμ-TCL1 mice at 2, 4, and 6 months of age. Splenocytes were stained with CD19-APC-Cy7, B220-Alexa 488, and CD5-APC and analyzed for B220+/CD5− B cells and B220^low^/CD5+ CLL cells on gated CD19+ populations. **B**, **C** CLL cells were examined for the expression of B220 in the peripheral blood (**B**) and spleens (**C**) of 6-month-old Eμ-TCL1 (*n* = 11) and AID^−/−^/Eμ-TCL1 (*n* = 11) mice. **D** CLL cells purified from the spleens of Eμ-TCL1 and AID^−/−^/Eμ-TCL1 mice were activated with anti-mouse IgM F(ab’)2 for the indicated times and lysed for immunoblot analyses of the indicated proteins. **E** CLL cells purified from the spleens of Eμ-TCL1 (*n* = 5) and AID^−/−^/Eμ-TCL1 (*n* = 6) mice were stimulated with 20 μg/mL LPS for 3 days and examined for phospho-SYK by flow cytometry. **F** CLL cells purified from the spleens of Eμ-TCL1 (*n* = 5) and AID^−/−^/Eμ-TCL1 (*n* = 6) mice were stimulated with 20 μg/mL LPS for 2 days, subsequently treated with 4 μM ibrutinib for 1 day, and subjected to XTT assays. Percentage growth was determined by comparing cells treated with ibrutinib with those that were untreated.

We next considered leukemia-intrinsic factors that could make AID-deficient CLL cells more malignant and sustain their survival. While examining the immune populations by flow cytometry, AID^−/−^/Eμ-TCL1 CLL cells were observed to have lower levels of B220, the CD45R isoform of the CD45 phosphatase, on the surface of CLL cells both in the peripheral blood and spleens of AID^−/−^/Eμ-TCL1 mice (Fig. 2A–C). CD45 had been shown to regulate BCR signaling [31], and dysregulation of CD45 expression could result in higher BCR signaling to sustain CLL growth. Thus, we proceeded to examine BCR signaling in CLL cells freshly purified from the spleens of AID^−/−^/Eμ-TCL1 mice. AID^−/−^/Eμ-TCL1 CLL cells had higher levels of BCR signaling, as shown by increased phosphorylation levels of Igα, spleen tyrosine kinase (SYK), and Bruton’s tyrosine kinase (BTK) upon activation with anti-mouse IgM F(ab’)2 as compared to AID-proficient Eμ-TCL1 CLL cells (Fig. 2D). AID-deficient CLL cells stimulated with LPS had higher levels of phospho-SYK as measured by flow cytometry (Fig. 2E) and were also more sensitive to treatment with a BTK inhibitor ibrutinib (Fig. 2F).

### AID deficiency results in upregulation of the IRE1/XBP1s pathway

To determine the molecular mechanisms behind the more malignant phenotypes of AID-deficient CLL cells in an unbiased manner, we performed RNA-sequencing on Eμ-TCL1 and AID^−/−^/Eμ-TCL1 CLL cells and analyzed the data by gene set enrichment analysis (GSEA). One of the most significantly upregulated pathways as determined by GSEA (*p* < 0.05, FDR < 0.1) was oxidative phosphorylation (Fig. S7A). Activation of oxidative phosphorylation in human patient CLL cells has been linked to the proliferative drive and clinical outcome of CLL [32]. We found that LPS- or CpG-1826-stimulated B cells from AID^−/−^/Eμ-TCL1 mice indeed produced significantly more ATP than those from Eμ-TCL1 mice (Fig. S7B, C).

**Fig. 3:**
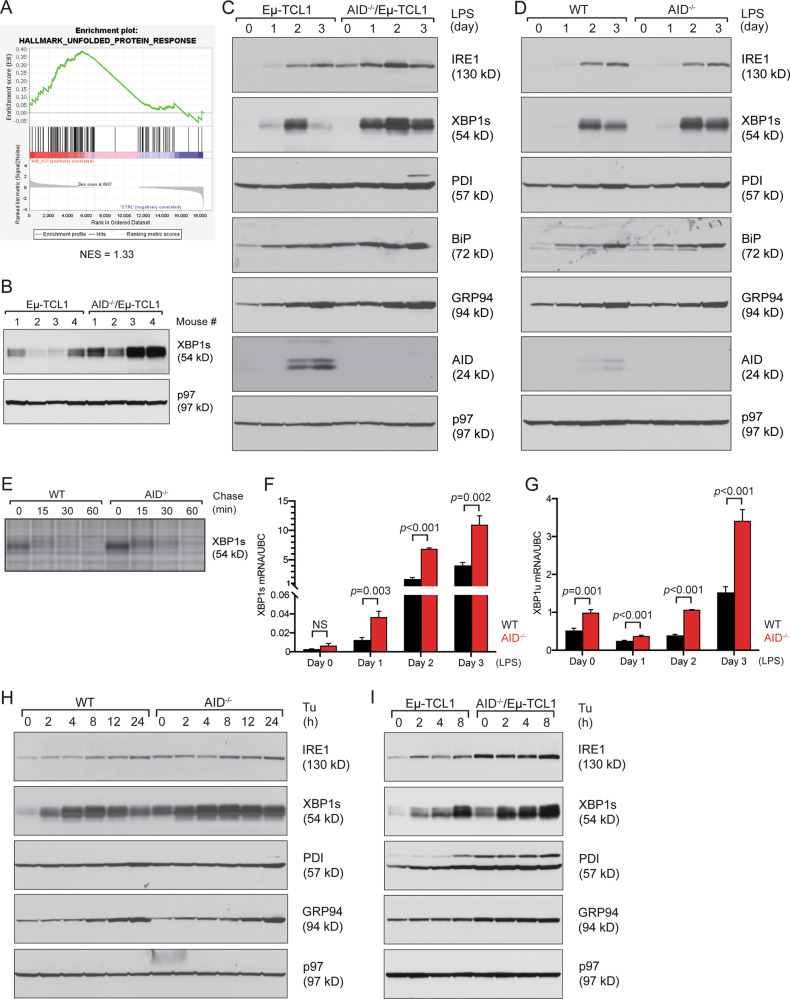
AID deficiency leads to an increased ER stress response in CLL cells and B cells, particularly through the IRE1/XBP1s pathway. **A** Gene set enrichment analysis from RNA sequencing of Eμ-TCL1 and AID^−/−^/Eμ-TCL1 CLL cells shows that mRNAs related to the unfolded protein response are upregulated in AID^−/−^/Eμ-TCL1 CLL cells. **B** CLL cells purified from the spleens of 4 Eμ-TCL1 and 4 AID^−/−^/Eμ-TCL1 mice were stimulated with 20 μg/mL LPS for 3 days and lysates were immunoblotted for the indicated proteins. **C** CLL cells purified from the spleens of Eμ-TCL1 and AID^−/−^/Eμ-TCL1 mice were stimulated with 20 μg/mL LPS for 3 days and lysates were immunoblotted for the indicated proteins. **D** B cells purified from the spleens of WT and AID^−/−^ mice were stimulated with 20 μg/mL LPS for 3 days and lysates were immunoblotted for the indicated proteins. **E** B cells purified from the spleens of WT and AID^−/−^ mice were stimulated with 20 μg/mL LPS for 3 days, starved in cysteine- and methionine-free medium for 1 h, radiolabeled for 20 min, and chased for the indicated times. Lysates were immunoprecipitated with an anti-XBP1s antibody and analyzed by SDS-PAGE and autoradiography. **F**–**G** B cells purified from the spleens of WT and AID^−/−^ mice were stimulated with 20 μg/mL LPS for 3 days and lysed for purification of RNA. The mRNA levels of XBP1s (**F**) and XBP1u (**G**) were measured by quantitative RT-PCR in triplicate. Data were normalized to UBC (a house-keeping gene) and shown as means ± SD. **H** B cells purified from the spleens of WT and AID^−/−^ mice were stimulated with 20 μg/mL LPS for 2 days and subsequently treated with 10 μg/mL tunicamycin for indicated times. Lysates were immunoblotted for the indicated proteins. **I** CLL cells purified from the spleens of Eμ-TCL1 and AID^−/−^/Eμ-TCL1 mice were stimulated with 20 μg/mL LPS for 2 days and subsequently treated with 10 μg/mL tunicamycin for indicated times. Lysates were immunoblotted for the indicated proteins.

Another highly significantly upregulated pathway as determined by GSEA (*p* < 0.05, FDR < 0.1) was the unfolded protein response (Fig. 3A). LPS-stimulated AID-deficient CLL cells were examined for the expression of ER stress response proteins and exhibited higher levels of IRE1, XBP1s, protein disulfide isomerase (PDI), and ER chaperones BiP and GRP94 (Fig. 3B, C). This increase in the ER stress response was also seen when AID-deficient CLL cells were stimulated with the TLR9 ligand CpG-1826 (Fig. S8A). To rule out that an accelerated tumor progression itself could cause an increase in the ER stress response, we next examined AID-deficient B cells in a non-cancer setting. AID^−/−^ B cells stimulated with LPS or CpG-1826 expressed similar levels of IRE1, PDI, BiP, and GRP94 as wild type (WT) B cells; however, they had significantly higher levels of XBP1s, indicating that the loss of AID alone results in increased XBP1s (Figs. 3D, S8B). As XBP1s has a short half-life, we next assessed the stability of XBP1s in AID^−/−^ B cells to determine if increased stability resulted in its accumulation. Radiolabeled pulse chase experiments followed by immunoprecipitation of XBP1s showed that AID^−/−^ B cells produced increased amounts of XBP1s that degraded at a similar rate to XBP1s produced in WT B cells (Fig. 3E). To identify if increased XBP1s production was transcriptionally controlled, we performed quantitative RT-PCR for spliced and unspliced XBP1 mRNA and found that both were higher in the AID^−/−^ B cells, indicating that AID deficiency causes increased transcription of XBP1s (Figs. 3F, G, S8C, D). We also looked at the other branches of the ER stress response to see if they were upregulated in AID^−/−^ B cells, but did not detect a significant difference in the expression of PERK, eIF2α, ATF4, and ATF6, indicating that the effect of AID on the ER stress response is primarily through the IRE1/XBP1s pathway (Fig. S8E).

### AID-deficient B cells and CLL cells are more responsive to ER stress

Because cancers often upregulate the ER stress response and overexpression of TCL1 in Eμ-TCL1 mice can activate this response, we wanted to investigate if AID deficiency resulted in an enhanced ER stress response. We treated B cells with the ER stressors tunicamycin (Tu), which inhibits protein glycosylation, thapsigargin (Tg), which inhibits the calcium transporter SERCA, and subtilase cytotoxin (SubAB), which cleaves BiP. AID^−/−^ B cells treated with Tu produced higher levels of IRE1 and XBP1s (Fig. 3H). This was also seen in AID^−/−^ B cells treated with Tg and SubAB, indicating that these cells are generally more responsive to ER stress than normal B cells (Fig. S9A, B). When examined for XBP1s by flow cytometry, Tu-, Tg-, and SubAB-treated AID^−/−^ B cells exhibited a higher percentage of XBP1s+ cells and increased expression levels of XBP1s (Figs. S9C–H, S10). AID^−/−^/Eμ-TCL1 CLL cells were also more responsive to ER stress induced with Tu, Tg, and SubAB than Eμ-TCL1 CLL cells (Figs. 3I, S11).

### AID-downregulated or IgHV-unmutated human CLL upregulates the expression of XBP1s

**Fig. 4:**
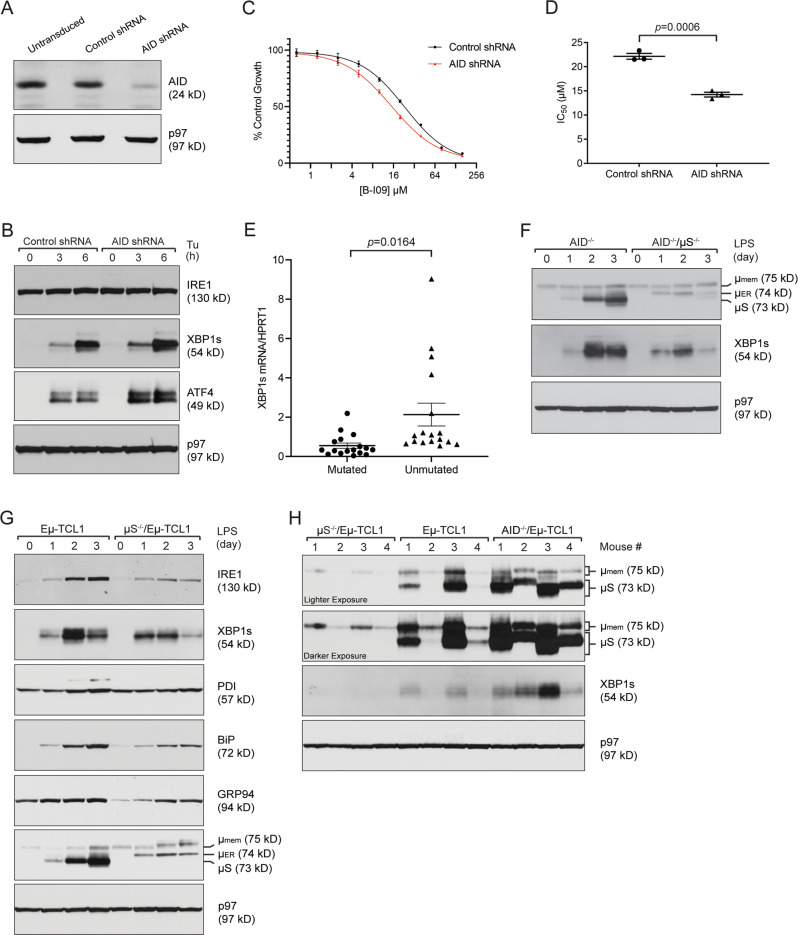
AID-downregulated human CLL cells upregulate the ER stress response and primary IgHV-unmutated human CLL cells express higher levels of XBP1s mRNA; loss of secretory IgM leads to downregulation of XBP1s production in CLL cells. **A** WaC3 human CLL cells were not transduced, transduced with a non-targeting control shRNA, or transduced with an AID-targeting shRNA, and lysates were immunoblotted for the indicated proteins. **B** Control shRNA- or AID-targeting shRNA-transduced WaC3 cells were treated with 10 μg/mL tunicamycin for the indicated times, and lysates were immunoblotted for the indicated proteins. **C** Control shRNA- or AID-targeting shRNA-transduced WaC3 cells were treated with indicated concentrations of B-I09 for 3 days and subjected to XTT assays. Percentage growth was determined by comparing cells treated with B-I09 with those that were untreated and are shown as the means ± SD. **D** IC_50_ values from three independent XTT experiments comparing control shRNA- or AID-targeting shRNA-transduced WaC3 cells treated with B-I09 for 3 days. **E** Primary human CLL cells purified from the peripheral blood of patients with IgHV-mutated (*n* = 17) and IgHV-unmutated (*n* = 17) CLL were lysed for purification of RNA. The mRNA levels of XBP1s were measured by quantitative RT-PCR and data were normalized to HPRT1 (a house-keeping gene). **F** B cells purified from the spleens of AID^−/−^ and AID^−/−^/μS^−/−^ mice were stimulated with 20 μg/mL LPS for 3 days and lysates were immunoblotted for the indicated proteins. **G** CLL cells purified from the spleens of Eμ-TCL1 and μS^−/−^/Eμ-TCL1 mice were stimulated with 20 μg/mL LPS for 3 days and lysates were immunoblotted for the indicated proteins. **H** CLL cells purified from the spleens of 4 μS^−/−^/Eμ-TCL1, 4 Eμ-TCL1, and 4 AID^−/−^/Eμ-TCL1 mice were directly lysed and immunoblotted for the indicated proteins.

Next, we wanted to confirm that the results found in AID-deficient Eμ-TCL1 mice were applicable to human CLL. We transduced the EBV-transformed human CLL cell line WaC3 with a control non-targeting shRNA and an AID-targeting shRNA and confirmed that AID expression was reduced in AID-targeting shRNA-transduced WaC3 cells (Fig. 4A). These AID-downregulated WaC3 cells had a higher ER stress response when treated with Tu, Tg, and SubAB, as shown by higher expression levels of XBP1s and ATF4 (Figs. 4B, S12). AID-targeting shRNA-transduced WaC3 cells were also more sensitive to killing by an inhibitor of the IRE1/XBP1s pathway, B-I09, with an average IC_50_ of 22.2 μM for the control shRNA-transduced cells compared to 14.2 μM for the AID-targeting shRNA-transduced cells (Fig. 4C, D). Moreover, we obtained primary CLL cells from the peripheral blood of patients with either IgHV-mutated or IgHV-unmutated CLL and determined that the more malignant IgHV-unmutated CLL cells had higher expression levels of XBP1s mRNA (Fig. 4E).

### Increased production of secretory IgM in AID-deficient CLL cells contributes to elevated levels of XBP1s

AID deficiency causes hyper-IgM syndrome in humans [5] and increased secretory IgM levels in mice (Figs. S2B, S6E). We hypothesized that these increased levels of secretory IgM, the largest antibody comprised of a pentamer of immunoglobulin molecules, would lead to upregulation of XBP1s to promote the protein folding capacity of the ER. To determine if secretory IgM levels could affect XBP1s production, we crossed μS^−/−^ mice, which are unable to produce secretory IgM [33], with AID^−/−^ mice to produce AID^−/−^/μS^−/−^ mice. AID^−/−^/μS^−/−^ B cells were unable to produce secretory IgM and consequently had lower expression levels of XBP1s when compared to AID^−/−^ B cells (Fig. 4F). In human CLL, IgM gammopathy has been linked to a more advanced stage of disease and a shorter treatment-free survival [34, 35]. We had previously generated μS^−/−^/Eμ-TCL1 mice and reported that these mice died significantly later than Eμ-TCL1 controls due to the reduced accumulation of MDSCs [28]. When further examining these mice, we found that secretory IgM-deficient CLL cells expressed lower levels of IRE1, XBP1s, BiP, and GRP94 in response to LPS stimulation, suggesting that these CLL cells are also intrinsically less malignant (Fig. 4G). When comparing μS^−/−^/Eμ-TCL1, Eμ-TCL1, and AID^−/−^/Eμ-TCL1 CLL cells freshly purified from the spleens of these mice, we found that although there was significant clonal variation between samples, AID-deficient CLL cells produced the most secretory IgM and XBP1s, while secretory IgM-deficient CLL cells produced the least XBP1s (Fig. 4H).

### AID^−/−^/Eμ-TCL1 CLL cells have altered Smad1/S1pr2 expression and migration

**Fig. 5:**
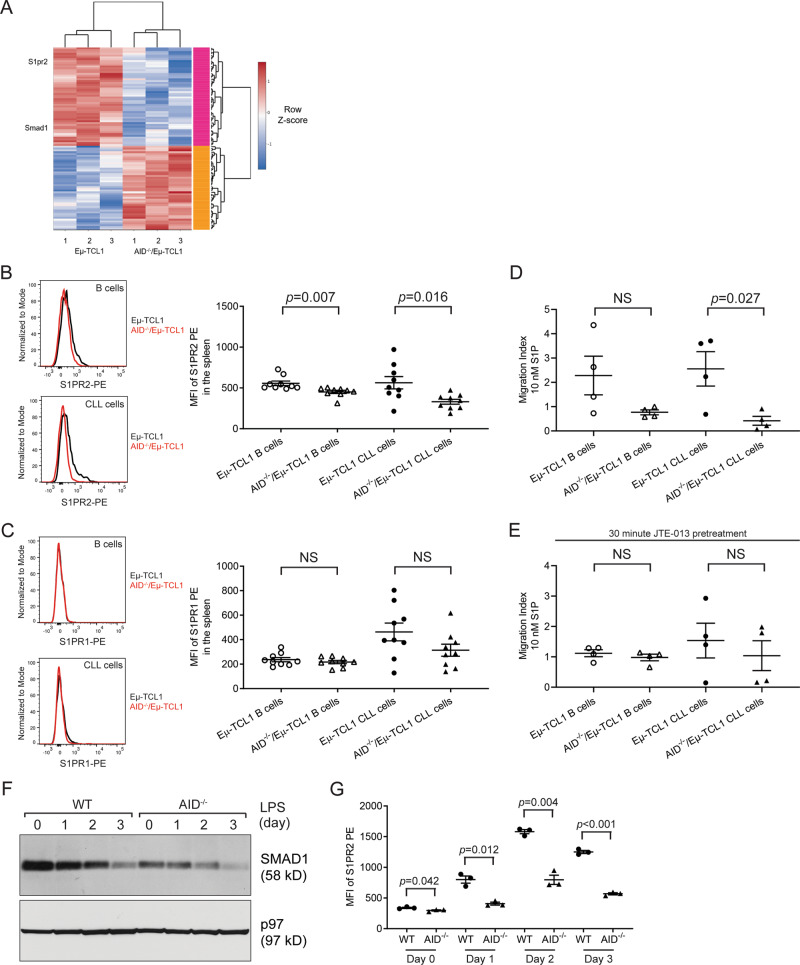
AID^−/−^ B cells and CLL cells have downregulated Smad1/S1pr2 and exhibit less migration in response to S1P. **A** A heat map of the top 100 differentially expressed genes from RNA sequencing of CLL cells from 3 Eμ-TCL1 mice and 3 AID^−/−^/Eμ-TCL1 mice shows that Smad1 and S1pr2 mRNAs are strongly downregulated in AID^−/−^/Eμ-TCL1 CLL cells compared to Eμ-TCL1 CLL cells. **B**, **C** B cells and CLL cells were examined for the expression of S1PR2 (**B**) and S1PR1 (**C**) in the spleens of 4-month-old Eμ-TCL1 (*n* = 9) and AID^−/−^/Eμ-TCL1 (*n* = 9) mice by flow cytometry. **D** Transwell assay of migration of B cells and CLL cells purified from the spleens of Eμ-TCL1 (*n* = 4) and AID^−/−^/Eμ-TCL1 (*n* = 4) mice towards 10 nM S1P. **E** B cells and CLL cells purified from the spleens of Eμ-TCL1 (*n* = 4) and AID^−/−^/Eμ-TCL1 (*n* = 4) mice were pretreated with 10 μM of the S1PR2 inhibitor JTE-013 for 30 min before the transwell migration assay towards 10 nM S1P. **F** B cells purified from the spleens of WT and AID^−/−^ mice were stimulated with 20 μg/mL LPS for 3 days and lysates were immunoblotted for the indicated proteins. **G** B cells purified from the spleens of WT and AID^−/−^ mice were stimulated with 20 μg/mL LPS for 3 days and examined for the expression of S1PR2 by flow cytometry.

The increased mortality of AID^−/−^/Eμ-TCL1 mice with no significant increase in the percentages of CLL cells in the peripheral blood and spleens prompted us to hypothesize that alterations in migration or homing signaling could lead to aberrant trafficking of CLL cells to other organs, resulting in AID^−/−^/Eμ-TCL1 mice dying earlier than their AID-proficient counterparts. When examining the RNA-sequencing data for transcriptional changes in migration- or homing-related mRNAs, we found that two strongly downregulated transcripts were those of Smad1 and S1pr2 (Fig. 5A). Smad1 is the transcriptional regulator of the sphingosine phosphate receptor S1pr2, and this tumor suppressive pathway is inactivated in B cell lymphoma [36]. S1pr2 deficiency in B cells leads to GC B cell proliferation and enlargement of the germinal center [37, 38], a phenotype that is also seen in AID deficiency (Fig. 1O). We performed flow cytometry analyses for S1PR2 and the related receptor S1PR1, which has been implicated as a factor in lymphocyte egress in CLL [39, 40]. Both B cells and CLL cells from the spleens of AID^−/−^/Eμ-TCL1 mice expressed lower levels of S1PR2 but not S1PR1 as measured by flow cytometry (Fig. 5B, C). Similar results were also seen in the peripheral blood of Eμ-TCL1 and AID^−/−^/Eμ-TCL1 mice (Fig. S13A, B).

To identify if these alterations in chemokine receptor expression were sufficient to affect cell migration, we performed transwell migration assays using the ligand for S1PR1 and S1PR2, sphingosine-1-phosphate (S1P). AID-deficient CLL cells migrated significantly less towards S1P than their AID-proficient counterparts, and although not statistically significant, AID^−/−^/Eμ-TCL1 B cells also responded towards S1P with less migration (Fig. 5D). To determine if this effect was through S1PR1 or S1PR2, we pretreated cells with the specific S1PR2 inhibitor JTE-013 for 30 min. After S1PR2 was inhibited, there was no longer a significant difference in migration, indicating that the initial difference in migration towards S1P was a result of S1PR2 expression (Fig. 5E). We also examined the expression of SMAD1 and S1PR2 in WT and AID^−/−^ B cells to see if the loss of AID affects this pathway directly. When compared to WT B cells, AID^−/−^ B cells had decreased expression of SMAD1 and correspondingly lower levels of S1PR2 but not S1PR1 (Figs. 5F, G, S13C).

### Increased numbers of CLL cells from AID^−/−^/Eμ-TCL1 mice invaded the peritoneal cavity, lungs, and liver

**Fig. 6:**
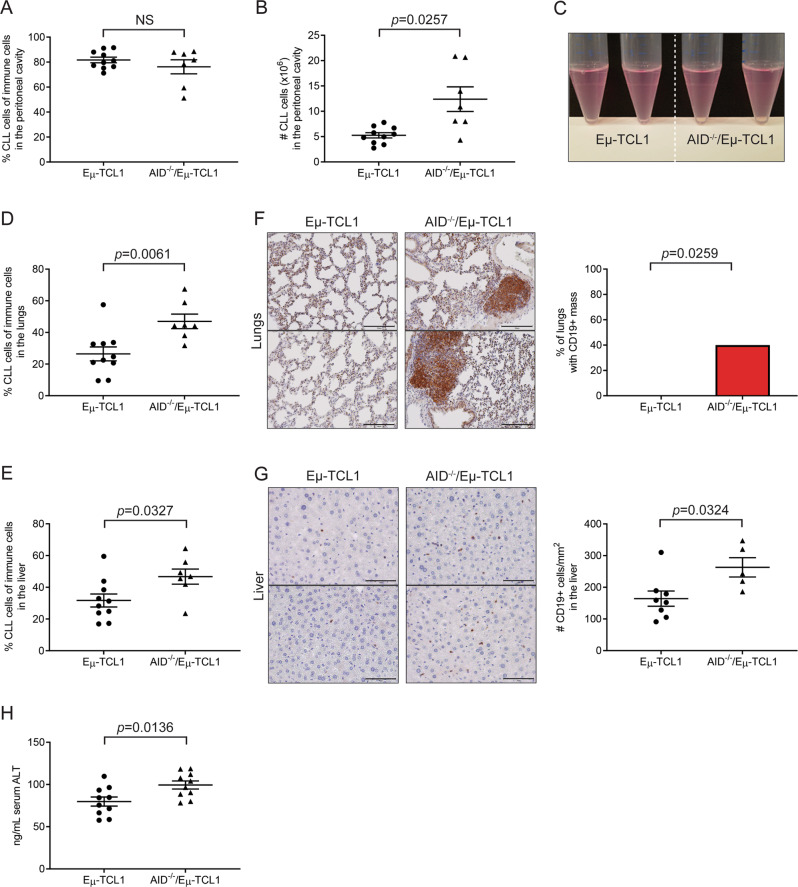
AID^−/−^/Eμ-TCL1 mice exhibited higher levels of CLL infiltration into the peritoneal cavity, lungs, and liver. **A**, **B** Quantification of percentages (**A**) and numbers (**B**) of CLL cells in the peritoneal cavities of 6-month-old Eμ-TCL1 (*n* = 10) and AID^−/−^/Eμ-TCL1 (*n* = 7) mice by flow cytometry and cell counting. Peritoneal cavity single cell suspensions were analyzed for CD19+/B220^low^/CD5+ CLL cells on the gated immune cell populations as determined by positive expression of CD19, CD3, or CD11b. **C** Representative images of cells collected from the peritoneal cavities of 2 Eμ-TCL1 and 2 AID^−/−^/Eμ-TCL1 mice at 6 months of age. **D** Quantification of CLL cells in the lungs of 6-month-old Eμ-TCL1 (*n* = 10) and AID^−/−^/Eμ-TCL1 (*n* = 7) mice by flow cytometry. Lung single cell suspensions were analyzed for CD19+/B220^low^/CD5+ CLL cells on the gated immune cell populations as determined by positive expression of CD19, CD3, or CD11b. **E** Quantification of CLL cells in the livers of 6-month-old Eμ-TCL1 (*n* = 10) and AID^−/−^/Eμ-TCL1 (*n* = 7) mice by flow cytometry. Liver single cell suspensions were analyzed for CD19+/B220^low^/CD5+ CLL cells on the gated immune cell populations as determined by positive expression of CD19, CD3, or CD11b. **F** Representative images and quantification of CD19+ masses in lung sections from 6-month-old Eμ-TCL1 (*n* = 8) and AID^−/−^/Eμ-TCL1 (*n* = 5) mice by immunohistochemistry. Scale bars: 150 μm. **G** Representative images and quantification of CD19+ cells in liver sections from 6-month-old Eμ-TCL1 (*n* = 8) and AID^−/−^/Eμ-TCL1 (*n* = 5) mice by immunohistochemistry. Scale bars: 75 μm. **H** Serum levels of ALT in 6-month-old Eμ-TCL1 (*n* = 10) and AID^−/−^/Eμ-TCL1 (*n* = 10) mice were determined by ELISA.

We then sought to confirm that CLL cells were being found in the other organs of AID^−/−^/Eμ-TCL1 mice, which could lead to their premature deaths compared to Eμ-TCL1 mice. 6-month-old Eμ-TCL1 and AID^−/−^/Eμ-TCL1 mice were sent for pathological examinations, and the pathology report found that there were increased levels of lymphocyte infiltration in the lungs and livers of AID^−/−^/Eμ-TCL1 mice compared to Eμ-TCL1 mice. To follow up on these preliminary studies, we took 6-month-old Eμ-TCL1 and AID^−/−^/Eμ-TCL1 mice and performed flow cytometry and immunohistochemistry to investigate if these infiltrates were leukemic. The peritoneal cavities of AID^−/−^/Eμ-TCL1 mice, while not containing a higher percentage of CLL cells, did have greater numbers of these cells (Fig. 6A–C). Flow cytometry analyses showed that the lungs and livers of AID^−/−^/Eμ-TCL1 mice had significantly higher percentages of CLL cells (Fig. 6D, E). Immunohistochemistry also confirmed that the lungs and livers had more infiltration of CD19+ cells (Fig. 6F, G). Several of the lungs in AID^−/−^/Eμ-TCL1 mice also had lymphomatous masses comprised of CD19+ cells which were not seen in Eμ-TCL1 mice (Fig. 6F). With increased leukemic infiltration into the livers, AID^−/−^/Eμ-TCL1 mice correspondingly exhibited higher levels of serum alanine transaminase (ALT), a marker of liver damage (Fig. 6H). There was not a significant difference in percentages of CLL cells in the bone marrow or lymph nodes of AID^−/−^/Eμ-TCL1 mice when compared to those of Eμ-TCL1 mice (Fig. S14).

### Combinations of an S1PR2 agonist and an IRE1 inhibitor are effective in killing CLL

**Fig. 7:**
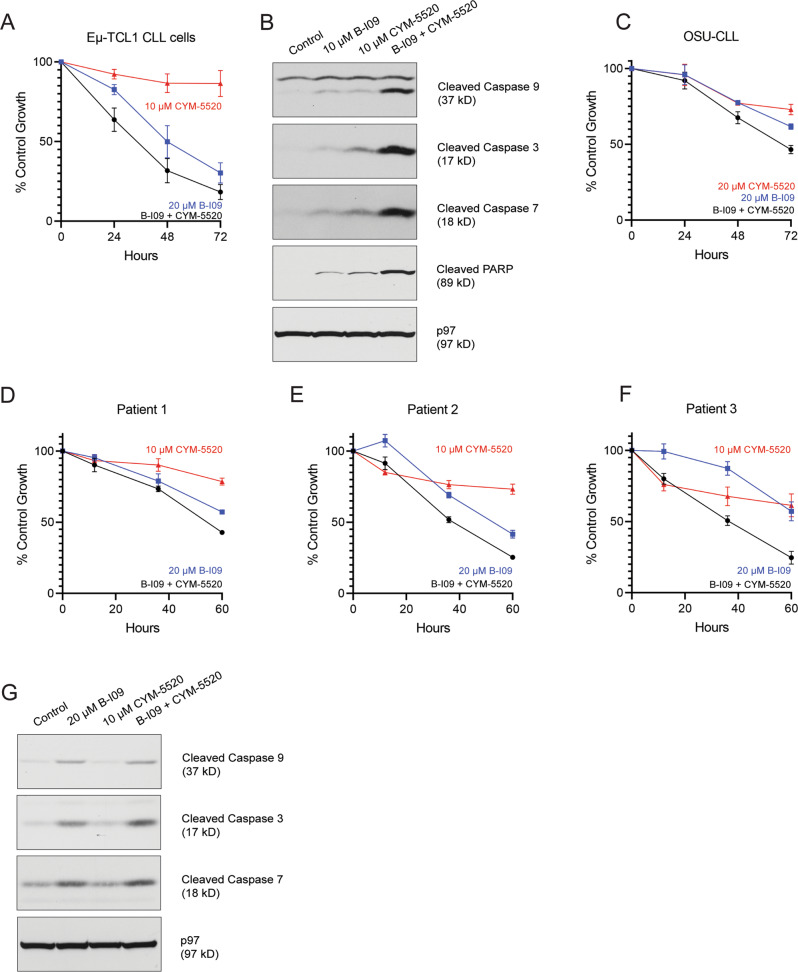
Combinations of the S1PR2 agonist CYM-5520 and the IRE1 inhibitor B-I09 show improved killing activity against primary mouse and human CLL cells. **A** CLL cells purified from the spleens of Eμ-TCL1 mice (*n* = 4) were treated with 20 μM B-I09, 10 μM CYM-5520, or the combination of both for 3 days and subjected to XTT assays. Percentages of growth were determined by comparing drug-treated groups with control groups and data are shown as means ± SEM. **B** CLL cells purified from the spleens of Eμ-TCL1 mice were treated with 10 μM B-I09, 10 μM CYM-5520, or the combination of both for 24 h and lysates were immunoblotted for the indicated proteins. **C** The human OSU-CLL cell line was treated with 20 μM B-I09, 20 μM CYM-5520, or the combination of both for 3 days and subjected to XTT assays. Percentages of growth were determined by comparing drug-treated groups with control groups and data are shown as means ± SD. **D**–**F** Primary human CLL cells purified from the peripheral blood of 3 patients were treated with 20 μM B-I09, 10 μM CYM-5520, or the combination of both for 60 hours and subjected to XTT assays. Percentages of growth were determined by comparing drug-treated groups with control groups and data are shown as means ± SD. **G** Primary human CLL cells purified from the peripheral blood were treated with 20 μM B-I09, 10 μM CYM-5520, or the combination of both for 24 hours and lysates were immunoblotted for the indicated proteins.

Because the altered expression of the SMAD1/S1PR2 and IRE1/XBP1s pathways could contribute to malignant progression of CLL, we tested whether they could be targeted together to deliver enhanced cytotoxicity against CLL. To target the tumor suppressive SMAD1/S1PR2 pathway and the leukemia-supporting IRE1/XBP1s pathway, we used the S1PR2 agonist CYM-5520 and the IRE1 inhibitor B-I09, respectively. CLL cells from Eμ-TCL1 mice were treated for 24 h with 20 μM B-I09, 10 μM CYM-5520, or the combination of both, and compared to the untreated control (Fig. 7A). Although 10 μM CYM-5520 had little effect on inhibiting the growth of CLL cells, it enhanced the growth inhibitory effect of B-I09. This growth inhibition was a result of apoptosis, as shown by increased proteolytic cleavage of caspases 9, 3, and 7, and PARP in the combination-treated CLL cells (Fig. 7B). The human CLL cell line OSU-CLL was also more sensitive to the combination of 20 μM B-I09 and 20 μM CYM-5520 compared to either agent alone (Fig. 7C). Lastly, we treated primary CLL cells from human patients with 20 μM B-I09, 10 μM CYM-5520, or the combination of both, and found that these CLL cells were inhibited the most by the combination (Fig. 7D–F). Growth inhibition in primary human CLL cells was also due to apoptosis, as evidenced by increased caspase cleavage in the combination-treated cells after 24 hours (Fig. 7G).

## Discussion

Since AID only expresses in B cells during the GC reaction, we hypothesize that AID plays a role during the clonal development of CLL by suppressing the expression of XBP1s and compromising BCR signaling resulting in the less aggressive phenotypes of IgHV-mutated CLL. Our results are distinct from, and not in conflict, with the recent study in which AID overexpression has been shown to cause non-Ig mutations and more aggressive CLL [41]. In this study, Eμ-TCL1 mice with constitutive AID overexpression driven by the actin or Ig kappa promoter can acquire off-target mutations in oncogenes and tumor suppressors in CLL cells, leading to aggressive progression of CLL in these models. With the consideration that AID is not constitutively expressed at high levels in all stages of B cell development and differentiation, we believe that it is physiologically relevant to examine the role of AID in CLL using knockout mouse models. Another recent study by Schubert et al. compared mice transplanted with TCL1 and AID-deficient TCL1 CLL cells and reported that AID contributed to accelerated CLL progression [42]. However, they also found that AID knockout in the TCL1 mouse model on a C57BL6/J background did not lead to increased survival of these mice when compared to control mice. Different from their conclusion, we found that AID-deficient CLL-bearing mice survive significantly shorter than their AID-proficient counterparts and that AID-deficient CLL cells clearly exhibited more malignant phenotypes. While we have drawn a different conclusion, we note that Schubert et al. have also reported that mice transplanted with CLL cells expressing low levels of AID mRNA had a shorter survival than those transplanted with CLL cells expressing high levels of AID mRNA. Such results are consistent with our conclusion: AID deficiency in CLL can lead to a more malignant disease.

Overall, our and others’ results signify that AID may play different roles in regulating the severity of CLL during the initial development and potentially in later progression of this disease. Although research has been done to develop AID inhibitors and it has been suggested that an AID inhibitor can be used to treat B cell malignancies [43, 44], the exacerbation of CLL in AID^−/−^/Eμ-TCL1 mice suggests that these inhibitors could have the unintended effect of generating a more aggressive CLL. Instead, it may be beneficial to investigate how to selectively enhance the expression levels or activity of AID in CLL cells as a targeted therapeutic approach in driving CLL towards the indolent IgHV-mutated phenotype.

## Materials and methods

### Mice and study approval

WT, AID^−/−^, μS^−/−^, AID^−/−^/μS^−/−^, OT-1, Eμ-TCL1, AID^−/−^/Eμ-TCL1, μS^−/−^/Eμ-TCL1, MD4^+/−^/Eμ-TCL1, and AID^−/−^/MD4^+/−^/Eμ-TCL1 mice were maintained at our animal facility following guidelines provided by the Houston Methodist Research Institute Institutional Animal Care and Use Committee (IACUC). WT, AID^−/−^, μS^−/−^, AID^−/−^/μS^−/−^, and OT-1 mice were maintained on the C57BL/6 background. All strains carrying Eμ-TCL1 were maintained on the B6C3 background. All experiments using mice were performed following protocols approved by the Houston Methodist Research Institute IACUC. Further descriptions of the materials and methods can be found in the supplementary information.

### Patient samples

Primary human CLL cells were obtained from patients prior to treatment by Dr. Javier A. Pinilla-Ibarz at the H. Lee Moffitt Cancer Center and by Dr. Sai Ravi Pingali at the Houston Methodist Cancer Center. Samples were collected following the guidelines in the IRB protocols approved by the H. Lee Moffitt Cancer Center and the Houston Methodist Research Institute. Informed consent was obtained from each patient in accordance with the Declaration of Helsinki.

## Supplementary information

Supplementary Materials, Legends and Figures
