# Supplementary material for: Loss of AID exacerbates the malignant progression of CLL
Source: Leukemia. 2022 Aug 30;36(10):2430–42. doi: 10.1038/s41375-022-01663-5 (PMC9522595; doi:10.1038/s41375-022-01663-5)
Supplement: Supplementary file 1 — Supplementary Materials, Legends and Figures [file 41375_2022_1663_MOESM1_ESM.pdf]

Supplementary Materials and Methods

Statistics

Statistical significance ($p < 0.05$) was determined by unpaired two-tailed Student's *t* test using GraphPad Prism software version 9.2. Data were graphed as the means ± SEM except where otherwise noted. Immunoblot data were performed at least in triplicate. Sample sizes were chosen according to previously performed power analysis. Animals were not excluded or randomized according to pre-established criteria, and investigators were not blinded to group allocation during the experiments or analysis.

Flow cytometry analysis

Single cell suspensions from the spleen, bone marrow, peripheral blood, peripheral lymph nodes, and peritoneal cavity were generated following RBC lysis. Livers and lungs were perfused with phosphate-buffered saline (PBS), cut into pieces, treated with 2 mg/ml collagenase D (Sigma) in HBSS (Sigma) for 30 minutes at 37ºC, and mashed through cell strainers to generate single cell suspensions. Cells were blocked using fetal bovine serum (FBS, Sigma) and cell surface staining was performed by incubating cells at 4ºC for 30 min with fluorescence-conjugated anti-mouse antibodies. Intracellular staining for XBP1s and FOXP3 was achieved using the BD Biosciences transcription factor buffer set. Intracellular staining for phospho-SYK was achieved using the BD Biosciences phosflow buffer set. The following antibodies were purchased (clone; source): B220-Alexa 488 (RA3-6B2; Biolegend), CD5-APC (53-7.3; Biolegend), CD19-Alexa 647 (6D5; Biolegend), CD19-APC-Cy7 (6D5; Biolegend), IgM-PE-Cy7 (RMM-1; Biolegend), GL7-PE (GL7; Biolegend), AA4.1-PE-Cy7 (AA4.1; Biolegend), CD1d-PerCP-Cy5.5 (1B1; Biolegend), CD23-FITC (B3B4; Biolegend), CD3-BV605 (145-2C11; Biolegend), CD3-APC-Cy7 (145-2C11; Biolegend), CD4-BV605 (RM4-5; Biolegend), CD8-PE-Cy7 (53-6.7; Biolegend), CD25-PE (PC61; Biolegend), FOXP3-Alexa 647 (150D; Biolegend), CD11c-BV421 (N418; Biolegend), CD11b-PE (M1/70; Biolegend), Ly6C-Alexa 488 (HK1.4; Biolegend), Ly6G-Alexa 647 (1A8; Biolegend), S1PR1 (713412; R&D), S1PR2 (E-12; Santa Cruz), phospho-SYK (17A/P; BD Biosciences), and XBP1s-Alexa 647 (Q3-695; BD Biosciences). Hen egg lysozyme (HEL; Sigma) was conjugated to N-hydroxysuccimidyl esters of Alexa Fluor 568 (Molecular Probes) in PBS containing 100 mM $KHCO_3$ for 1 h at room temperature in the dark. After the

conjugates were dialyzed against PBS, the protein concentration and the degree of labeling were determined by measuring the absorbance of the protein at 280 nm and the absorbance of the dye at its absorbance maximum. Viability staining was accomplished using DAPI or Fixable Viability Stain 450 (BD Biosciences). Acquisition of B cell populations was performed on an LSRII (BD Biosciences) or FACSymphony A3 (BD Biosciences). Cytometry data were analyzed using FlowJo software version 10.8.

Purification of mouse B cells and CLL cells

Splenocytes were obtained from mice by mashing the spleens through cell strainers followed by RBC lysis. Mouse B cells and CLL cells were purified from mouse spleens by negative selection using CD43 or Pan-B magnetic beads (Miltenyi Biotech), respectively, according to the manufacturer's instructions.

Cell culture

Mouse B cells, mouse CLL cells, human primary CLL cells, and human CLL cell lines were cultured in RPMI 1640 medium (Gibco) supplemented with 10% heat-inactivated FBS, 2 mM L-glutamine (Sigma), 100 U/ml penicillin (Gibco), 100 μg/ml streptomycin (Gibco), 1 mM sodium pyruvate (Gibco), 0.1 mM MEM non-essential amino acids (Gibco), and 0.1 mM 2-mercaptoethanol (2-ME, Sigma). WaC3 cells were obtained from the laboratory of Dr. Javier A. Pinilla-Ibarz at the Moffitt Cancer Center, Tampa, FL. OSU-CLL cells were a kind gift of Dr. John C. Byrd at the University of Cincinnati College of Medicine, Cincinnati, OH. All cell lines are authenticated and negative for mycoplasma contamination.

Antibodies and reagents

Polyclonal antibodies against Igα, PDI, and BiP were generated in rabbits. The following antibodies were purchased (source #catalog): AID (Cell Signaling #4949), p97 (Fitzgerald #10R-P104A), phospho-Igα (Cell Signaling #5173), phospho-SYK (Cell Signaling #2711), SYK (Cell Signaling #2712), phospho-BTK (Cell Signaling #5082), BTK (Cell Signaling #8547), XBP1s (Cell Signaling #12782), IRE1 (Cell Signaling #3294), BiP (Cell Signaling #3183), GRP94 (Cell Signaling #20292), GRP94 (Enzo Life Sciences #ADI-SPA-850), PERK (Cell Signaling #3192), eIF2α (Cell Signaling #2103), ATF4 (Cell Signaling #11815), IgM (SouthernBiotech

#1020-01), ATF6 (Proteintech #66563-1-Ig), SMAD1 (Cell Signaling #6944), mouse cleaved caspase 9 (Cell Signaling #9509), human cleaved caspase 9 (Cell Signaling #9501), cleaved caspase 3 (Cell Signaling #9664), cleaved caspase 7 (Cell Signaling #9491), and mouse cleaved PARP (Cell Signaling #9548). Sphingosine-1-phosphate (S1P; Sigma), lipopolysaccharide (LPS; Sigma), CpG-1826 (TIB-Molbiol), SIINFEKL (AnaSpec), CYM-5520 (Cayman), JTE-013 (Sigma), tunicamycin (Enzo Life Sciences), and thapsigargin (Enzo Life Sciences) were obtained commercially. SubAB was provided to us by Dr. James C. Paton and Dr. Adrienne W. Paton at the University of Adelaide, Adelaide, Australia. B-I09 and ibrutinib were chemically synthesized as described previously [1].

Immunoblotting

Cells were lysed in RIPA buffer (10 mM Tris-HCl, pH 7.4; 150 mM NaCl; 1% NP-40; 0.5% sodium deoxycholate; 0.1% SDS; 1 mM EDTA) supplemented with protease inhibitors (Roche) and phosphatase inhibitors. Protein concentrations were determined by BCA assay (Pierce). Proteins were boiled in SDS-PAGE sample buffer (62.5 mM Tris-HCl, pH 6.8; 2% SDS; 10% glycerol; 0.1% bromophenol blue) containing 2-ME, analyzed by SDS-PAGE, and transferred to nitrocellulose membranes. Membranes were blocked in 5% non-fat milk (wt/vol in PBS) and immunoblotted with indicated primary antibodies and appropriate HRP-conjugated secondary antibodies. Immunoblots were developed using the Western Lighting Chemiluminescence Reagent (PerkinElmer). The AAA-ATPase p97 was used as a loading control.

ELISA

For measurement of serum Ig, high binding assay plates (Corning) were coated with 2.5 µg/ml anti-Ig capture antibody (SouthernBiotech). IgM, IgG, and IgA in mouse serum were measured using horseradish peroxidase (HRP)-conjugated secondary antibodies against each mouse antibody isotype (SouthernBiotech) and 3,3′,5,5′-tetramethylbenzidine substrate (Sigma). After incubation for 15 minutes, the ELISA reaction was stopped using 1 M HCl and quantified at 450 nm using a BioTek Cytation 5 microplate reader. For measurement of serum ALT, the Mouse ALT ELISA Kit (Abcam) was used according to the manufacturer’s instructions.

Complete blood count

Complete blood counts were obtained using a Heska HemaTrue analyzer.

MDSC-mediated T cell suppression assays

MDSCs were purified from the spleens of CLL-bearing mice by positive selection using Ly6G magnetic beads (Miltenyi Biotech) according to the manufacturer's instructions. CD8+ T cells from OT-1 mice recognize ovalbumin peptides and respond with proliferation. Splenocytes from OT-1 mice were mixed with splenocytes from naïve mice in complete RPMI medium and plated into 96-well U-bottom plates with the ovalbumin SIINFEKL peptide and Ly6G+ MDSCs. After incubation for 48 hours, cells were radiolabeled with ^{3}H-thymidine (GE Healthcare) for 6 hours. The uptake of ^{3}H-thymidine was measured as counts per minute using a liquid scintillation counter and the percentage of proliferation in comparison to positive controls (responder cells with peptide without MDSCs) was calculated.

BCR activation

Mouse B cells were suspended in serum-free RPMI medium supplemented with 25 mM HEPES, stimulated with F(ab')2 fragments of anti-mouse IgM antibodies (20 μg/mL, SouthernBiotech) for indicated times, and lysed immediately by adding ice-cold lysis buffer (50 mM Tris-HCl, pH 8.0; 150 mM NaCl; 1% Triton X-100; 1 mM EDTA) supplemented with protease inhibitor cocktail (Roche), 4 mM sodium pyrophosphate, 2 mM sodium vanadate, and 10 mM sodium fluoride. Lysates were analyzed by SDS-PAGE and immunoblotted for molecules of interest.

Cell proliferation XTT assays

Appropriate numbers of cells were suspended in phenol red-free complete RPMI culture medium, seeded in 96-well cell culture plates, and treated with compounds for the indicated times. XTT assays (Roche) were performed according to the manufacturer's instructions. Following the addition of 50 μL XTT labeling reagent and 1 μL electron-coupling reagent to each well, cells were incubated for 4 hours in a CO_2 incubator to allow for the yellow

tetrazolium salt XTT to be cleaved by mitochondrial dehydrogenases of metabolically active cells. The resultant orange formazan dye was quantified at 492 nm using a BioTek Cytation 5 microplate reader.

RNA sequencing

Total RNA was isolated with TRIzol (Invitrogen). Illumina compatible libraries were generated using the QuantSeq 3' mRNA-Seq Library Prep Kit (Lexogen) and sequenced with an Illumina Nextseq 500. Data were analyzed using the integrated data analysis pipeline on the BlueBee Genomics platform and GSEA software [2].

ATP production assays

The XF Real-Time ATP Rate Assay (Agilent) was performed on an Agilent Seahorse XFe96 Analyzer according to the manufacturer's instructions and data were analyzed using Agilent Wave software.

Pulse chase and immunoprecipitation

Cells were starved in methionine- and cysteine-free medium containing dialyzed FBS for 1 hour and pulse labeled with 250 µCi/ml [35S]-methionine and [35S]-cysteine (PerkinElmer) for 20 minutes. After labeling, cells were incubated in the chase medium containing 2.5 mM cold methionine and 0.5 mM cysteine. At the end of each chase interval, radiolabeled cells were lysed in RIPA buffer containing protease inhibitors. Pre-cleared lysates were incubated with antibodies against XBP1s together with protein G-sepharose beads (Sigma). Immunoprecipitated samples were boiled in SDS-PAGE sample buffer (62.5 mM Tris-HCl, pH 6.8; 2% SDS; 10% glycerol; 0.1% bromophenol blue) containing 2-ME, analyzed by SDS-PAGE, and visualized by autoradiography.

RT-PCR

Total RNA was isolated with TRIzol (Invitrogen). The cDNA was synthesized from RNA with Superscript II reverse transcriptase (Invitrogen). SYBR green master mix (Invitrogen) was used with the following sets of primers to detect mRNA levels in quantitative PCR: mouse XBP1s, 5'-GTCCATGGGAAGATGTTCTGG-3' and 5'-CTGAGTCCGAATCAGGTGCAG-3'; mouse XBP1u, 5'-GTCCATGGGAAGATGTTCTGG-3' and 5'-CAGCACTCAGACTATGTGCA-3'; mouse UBC, 5'-CAGCCGTATATCTTCCCAGACT-3' and 5'-

CTCAGAGGGATGCCAGTAATCTA-3'; human XBP1s, 5'-TGCTGAGTCCGCAGCAGGTG-3' and 5'-GCTGGCAGGCTCTGGGGAAG-3'; human HPRT1, 5'-ATGACCAGTCAACAGGGGAC-3' and 5'-TGCCTGACCAAGGAAAGCAA-3'.

Lentiviral transduction

shRNA-mediated knockdown of AID in WaC3 CLL cells was performed using the TRCN0000412426 lentivirus (Sigma) with pLKO.1-puro Non-Mammalian shRNA Control (Sigma) as the non-targeting control. Cells were transduced for 48 hours with 8 µg/mL polybrene (Sigma). AID knockdown WaC3 CLL cell clones were established via limiting dilution in the presence of 1 µg/mL puromycin.

Cell migration assays

Cells were suspended in migration medium consisting of serum-free RPMI medium with 1% BSA and 1% penicillin-streptomycin. Some cells were pretreated with the S1PR2 inhibitor JTE-013 for 30 minutes before being resuspended in fresh migration medium. Cells were suspended in 100 µL migration medium in the top chamber of a 24-well transwell plate (Corning), with the bottom chamber containing 10 nM S1P in 600 µL migration medium. Cells were allowed to migrate through the 5 µm transwell inserts for 3 hours, followed by collection and counting by timed flow cytometry. The migration index was calculated by dividing the percentage of migrated B cells or CLL cells by the percentage of that population among total cells.

Immunohistochemistry

Mouse tissues were fixed in 10% neutral buffered formalin overnight. Paraffin-embedded sections of mouse tissues were sectioned and stained for CD19 (Cell Signaling) overnight at 4ºC. Slide images were obtained with an Invitrogen EVOS M5000 Imaging System and quantified with Fiji software [3]. Statistical significance for proportion of lungs with CD19+ masses was determined by one-tailed chi-square test.

Supplementary Figure Legends

Figure S1. CLL cells developed in $AID^{-/-}/MD4^{+/-}$/Eμ-TCL1 mice fail to recognize HEL and these mice die at an earlier age than $MD4^{+/-}$/Eμ-TCL1 mice.

(A) Splenocytes from $AID^{-/-}/MD4^{+/-}$/Eμ-TCL1 mice were stained with CD3-BV605, IgM-PE-Cy7, B220-Alexa 488, CD5-APC, and HEL-Alexa 568. Gated CD3-/IgM+ populations were analyzed for B220+/CD5- B cells and $B220^{low}$/CD5+ CLL cells. B cells (red) and CLL cells (blue) were analyzed for their HEL-binding capability. **(B)** Kaplan-Meier survival analysis of $MD4^{+/-}$/Eμ-TCL1 and $AID^{-/-}/MD4^{+/-}$/Eμ-TCL1 mice. **(C-D)** Kaplan-Meier survival analysis of female (C) and male (D) $MD4^{+/-}$/Eμ-TCL1 and $AID^{-/-}/MD4^{+/-}$/Eμ-TCL1 mice. **(E)** B cells purified from the spleens of $MD4^{+/-}$/Eμ-TCL1 and $AID^{-/-}/MD4^{+/-}$/Eμ-TCL1 mice were stimulated with 20 μg/mL LPS for 3 days and lysates were immunoblotted for the indicated proteins.

Figure S2. $AID^{-/-}$/Eμ-TCL1 mice undergo normal leukemic progression and are unable to produce class-switched antibodies.

(A) Splenocytes from $AID^{-/-}$/Eμ-TCL1 mice were stained with CD3-BV605, CD19-APC-Cy7, B220-Alexa 488, and CD5-APC. Gated CD19+ populations were analyzed for B220+/CD5- B cells and $B220^{low}$/CD5+ CLL cells. **(B-D)** Serum levels of IgM (B), IgG (C), and IgA (D) in 2-month-old Eμ-TCL1 (n=6) and $AID^{-/-}$/Eμ-TCL1 (n=6) mice were determined by ELISA.

Figure S3. $AID^{-/-}$/Eμ-TCL1 mice have similar numbers of lymphocytes to Eμ-TCL1 mice but have lower numbers of monocytes and granulocytes in the peripheral blood.

(A-C) Quantification of lymphocytes (A), monocytes (B), and granulocytes (C) in the peripheral blood of Eμ-TCL1 (n=11) and $AID^{-/-}$/Eμ-TCL1 (n=10 at 2 months and n=11 at 4 and 6 months) mice at 2, 4, and 6 months of age by complete blood count.

Figure S4. Gating strategies for analyses of B cells and CLL cells.

(A) Splenocytes from 6-month-old Eμ-TCL1 and AID$^{-/-}$/Eμ-TCL1 mice were stained with CD19-APC-Cy7, B220-Alexa 488, and CD5-APC. Gated CD19+ populations were analyzed for B220+/CD5- B cells and B220^{low}/CD5+ CLL cells. **(B)** Splenocytes from 6-month-old Eμ-TCL1 and AID$^{-/-}$/Eμ-TCL1 mice were stained with CD19-Alexa 647, GL7-PE, AA4.1-PE-Cy7, CD1d-PerCP-Cy5.5, and CD23-FITC. Gated CD19+ populations were analyzed for GL7+ activated B cells. Gated CD19+/GL7-/AA4.1- populations were analyzed for CD1d-/CD23+ follicular B cells and CD1d+/CD23- marginal zone B cells.

Figure S5. Gating strategies for analyses of T cells and T cell populations.

(A) Splenocytes from 6-month-old Eμ-TCL1 and AID$^{-/-}$/Eμ-TCL1 mice were stained with CD3-APC-Cy7, B220-Alexa 488, CD4-BV605, and CD8-PE-Cy7. Gated CD3+ populations were analyzed for CD8+ cytotoxic T cells and CD4+ helper T cells. **(B-D)** Quantification of CD3+ T cells (B), CD8+ cytotoxic T cells (C), and CD4+ helper T cells (D) in the spleens of 6-month-old Eμ-TCL1 (n=11) and AID$^{-/-}$/Eμ-TCL1 (n=11) mice by flow cytometry. **(E)** Splenocytes from 4-month-old Eμ-TCL1 and AID$^{-/-}$/Eμ-TCL1 mice were stained with CD3-APC-Cy7, CD4-BV605, FOXP3-Alexa 647, and CD25-PE and analyzed for CD3+/CD4+/FOXP3+/CD25+ regulatory T cells. **(F)** Quantification of regulatory T cells in the spleens of 4-month-old Eμ-TCL1 (n=3) and AID$^{-/-}$/Eμ-TCL1 (n=5) mice by flow cytometry.

Figure S6. Gating strategies for analyses of myeloid cells, myeloid cell populations, and MDSC suppression.

(A) Splenocytes from 6-month-old Eμ-TCL1 and AID$^{-/-}$/Eμ-TCL1 mice were stained with CD11c-BV421, CD11b-PE, Ly6C-Alexa 488, and Ly6G-Alexa 647. CD11c+ populations are dendritic cells and gated CD11c-/CD11b+ myeloid populations were analyzed for Ly6C^{intermediate}/Ly6G+ granulocytes and Ly6C^{+}/Ly6G- monocytes. **(B-D)** Quantification of granulocytes (B), monocytes (C), and dendritic cells (D) in the spleens of 6-month-old Eμ-TCL1 (n=11) and AID$^{-/-}$/Eμ-TCL1 (n=11) mice by flow cytometry. **(E)** Serum levels of IgM in 6-month-old Eμ-TCL1 (n=11) and AID$^{-/-}$/Eμ-TCL1 (n=11) mice were determined by ELISA. **(F)** Mixed OT-1/naïve splenocytes were incubated with granulocytes purified from spleens of Eμ-TCL1 (n=11) and AID$^{-/-}$/Eμ-TCL1 (n=11) mice in the presence of the SIINFEKL peptide for 48 hours and subsequently radiolabeled with ^{3}H-thymidine for 6 hours.

Percentage proliferation was determined by comparing splenocytes cultured with MDSCs with those that were cultured alone.

Figure S7. AID deficiency leads to an upregulation in oxidative phosphorylation and ATP production.

(A) Gene set enrichment analysis from RNA sequencing of Eμ-TCL1 and $AID^{-/-}$/Eμ-TCL1 CLL cells shows that mRNAs related to oxidative phosphorylation are upregulated in $AID^{-/-}$/Eμ-TCL1 CLL cells. **(B-C)** B cells purified from the spleens of 2-month-old Eμ-TCL1 and $AID^{-/-}$/Eμ-TCL1 mice were stimulated with 20 μg/mL LPS (B) or 0.5 μM CpG-1826 (C) for 2 days and analyzed for ATP production.

Figure S8. AID deficiency leads to an increased ER stress response in CLL cells and B cells in response to CpG-1826 treatment but does not alter the other branches of the ER stress response.

(A) CLL cells purified from the spleens of Eμ-TCL1 and $AID^{-/-}$/Eμ-TCL1 mice were stimulated with 0.5 μM CpG-1826 for 3 days and lysates were immunoblotted for the indicated proteins. **(B)** B cells purified from the spleens of WT and $AID^{-/-}$ mice were stimulated with 0.5 μM CpG-1826 for 3 days and lysates were immunoblotted for the indicated proteins. **(C-D)** B cells purified from the spleens of WT and $AID^{-/-}$ mice were stimulated with 0.5 μM CpG-1826 for 3 days and lysed for purification of RNA. The mRNA levels of XBP1s (C) and XBP1u (D) were measured by quantitative RT-PCR in triplicate. Data were normalized to UBC (a house-keeping gene) and shown as means ± SD. **(E)** B cells purified from the spleens of WT and $AID^{-/-}$ mice were stimulated with 20 μg/mL LPS for 3 days and lysates were immunoblotted for the indicated proteins.

Figure S9. AID-deficient B cells are more responsive to ER stress.

(A-B) B cells purified from the spleens of WT and $AID^{-/-}$ mice were stimulated with 20 μg/mL LPS for 2 days and subsequently treated with 2.5 μM thapsigargin (A) or 100 ng/mL SubAB (B) for indicated times. Lysates were immunoblotted for the indicated proteins. **(C-H)** B cells purified from the spleens of WT and $AID^{-/-}$ mice were stimulated with 20 μg/mL LPS for 1 day, subsequently treated with 10 μg/mL tunicamycin (C-D), 2.5 μM thapsigargin (E-F), or 100 ng/mL SubAB (G-H) for indicated times, and examined by flow cytometry for percentages of cells expressing XBP1s and the MFI of XBP1s.

Figure S10. Representative flow cytometry plots for XBP1s in WT and AID$^{-/-}$ B cells after treatment with tunicamycin, thapsigargin, and SubAB.

(A-C) B cells purified from the spleens of WT and AID$^{-/-}$ mice were stimulated with 20 μg/mL LPS for 1 day, and subsequently treated with 10 μg/mL tunicamycin (A), 2.5 μM thapsigargin (B), or 100 ng/mL SubAB (C) for indicated times. B220+ B cells were examined by flow cytometry for the expression of XBP1s.

Figure S11. AID-deficient CLL cells are more responsive to ER stress.

(A-B) CLL cells purified from the spleens of Eμ-TCL1 and AID$^{-/-}$/Eμ-TCL mice were stimulated with 20 μg/mL LPS for 2 days and subsequently treated with 2.5 μM thapsigargin (A) or 100 ng/mL SubAB (B) for indicated times. Lysates were immunoblotted for the indicated proteins.

Figure S12. AID-downregulated WaC3 CLL cells are more responsive to ER stress.

(A-B) Control shRNA- or AID-targeting shRNA-transduced WaC3 cells were treated with 2.5 μM thapsigargin (A) or 100 ng/mL SubAB (B) for the indicated times. Lysates were immunoblotted for the indicated proteins.

Figure S13. AID deficiency results in altered S1PR2 but not S1PR1 expression in B cells and CLL cells.

(A-B) B cells and CLL cells in the peripheral blood of 4-month-old Eμ-TCL1 (n=9) and AID$^{-/-}$/Eμ-TCL1 (n=9) mice were examined for the expression of S1PR2 (A) and S1PR1 (B) by flow cytometry. **(C)** B cells purified from the spleens of WT and AID$^{-/-}$ mice were stimulated with 20 μg/mL LPS for 3 days and examined for the expression of S1PR1 by flow cytometry.

Figure S14. Eμ-TCL1 and AID$^{-/-}$/Eμ-TCL1 mice had similar levels of CLL cells in the bone marrow and lymph nodes.

(A) Quantification of CLL cells in the bone marrow of 6-month-old Eμ-TCL1 (n=11) and AID$^{-/-}$/Eμ-TCL1 (n=11) mice by flow cytometry. Bone marrow single cell suspensions were analyzed for CD19+/B220^{low}/CD5+ CLL cells on the gated immune cell populations as determined by positive expression of CD19, CD3, or CD11b. **(B)**

Quantification of CLL cells in the peripheral lymph nodes of 6-month-old Eμ-TCL1 (n=8) and $AID^{-/-}$/Eμ-TCL1 (n=8) mice by flow cytometry. Lymph node single cell suspensions were analyzed for CD19+/$B220^{low}$/CD5+ CLL cells on the gated immune cell populations as determined by positive expression of CD19, CD3, or CD11b.

References

1. Tang CH, Ranatunga S, Kriss CL, Cubitt CL, Tao J, Pinilla-Ibarz JA*, et al.* Inhibition of ER stress-associated IRE-1/XBP-1 pathway reduces leukemic cell survival. *J Clin Invest* 2014 Jun; **124**(6)**:** 2585-2598.

2. Subramanian A, Tamayo P, Mootha VK, Mukherjee S, Ebert BL, Gillette MA*, et al.* Gene set enrichment analysis: A knowledge-based approach for interpreting genome-wide expression profiles. *Proceedings of the National Academy of Sciences* 2005; **102**(43)**:** 15545-15550.

3. Schindelin J, Arganda-Carreras I, Frise E, Kaynig V, Longair M, Pietzsch T*, et al.* Fiji: an open-source platform for biological-image analysis. *Nature Methods* 2012 2012/07/01; **9**(7)**:** 676-682.

Figure S1

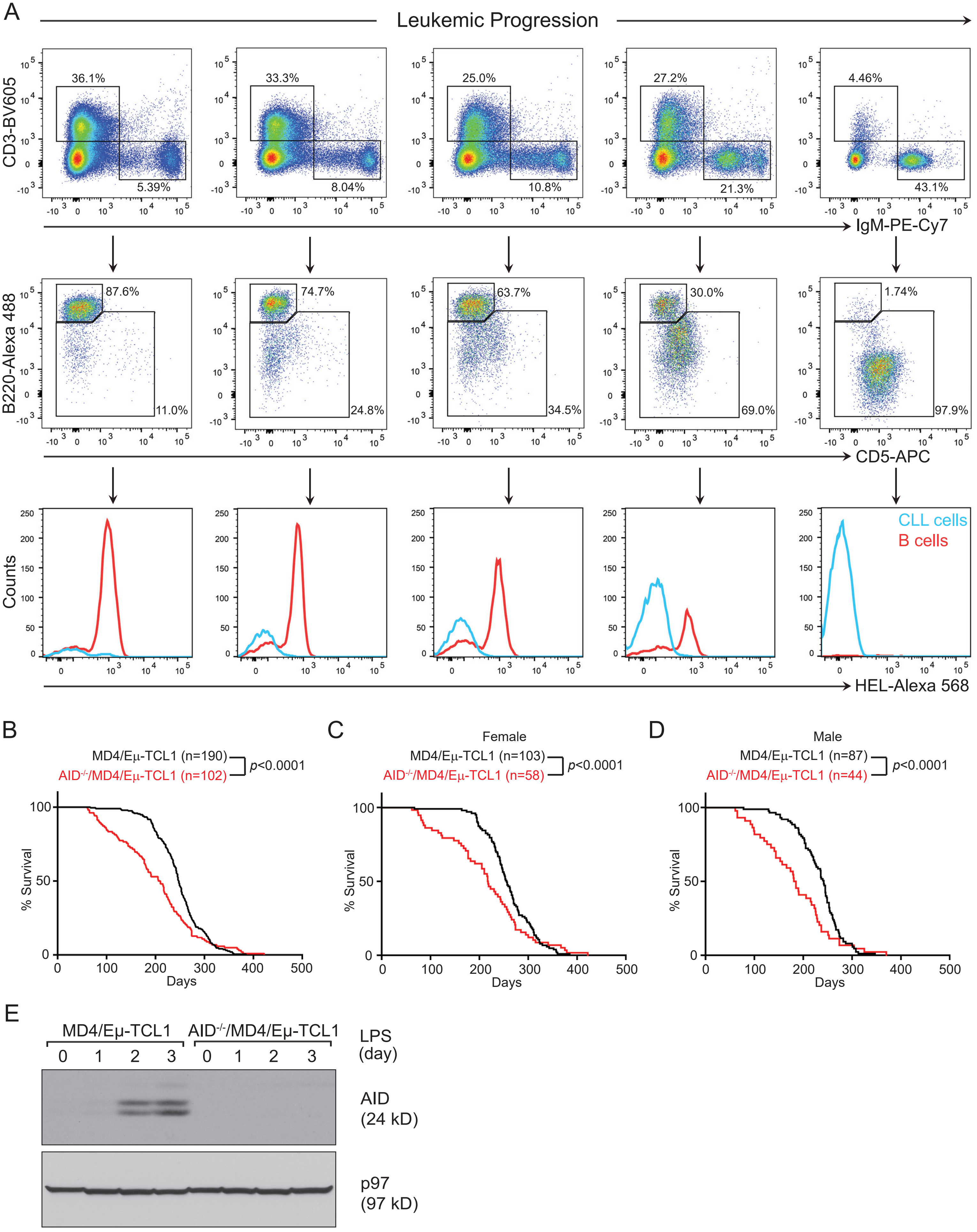
A
Leukemic Progression
CD3-BV605
36.1%
5.39%
33.3%
8.04%
25.0%
10.8%
27.2%
21.3%
4.46%
43.1%
IgM-PE-Cy7
B220-Alexa 488
87.6%
11.0%
74.7%
24.8%
63.7%
34.5%
30.0%
69.0%
1.74%
97.9%
CD5-APC
Counts
CLL cells
B cells
HEL-Alexa 568
B
MD4/Eμ-TCL1 (n=190)
AID-/-/MD4/Eμ-TCL1 (n=102)
p<0.0001
% Survival
Days
C
Female
MD4/Eμ-TCL1 (n=103)
AID-/-/MD4/Eμ-TCL1 (n=58)
p<0.0001
% Survival
Days
D
Male
MD4/Eμ-TCL1 (n=87)
AID-/-/MD4/Eμ-TCL1 (n=44)
p<0.0001
% Survival
Days
E
MD4/Eμ-TCL1
AID-/-/MD4/Eμ-TCL1
0 1 2 3 0 1 2 3
LPS (day)
AID (24 kD)
p97 (97 kD)

Figure S2

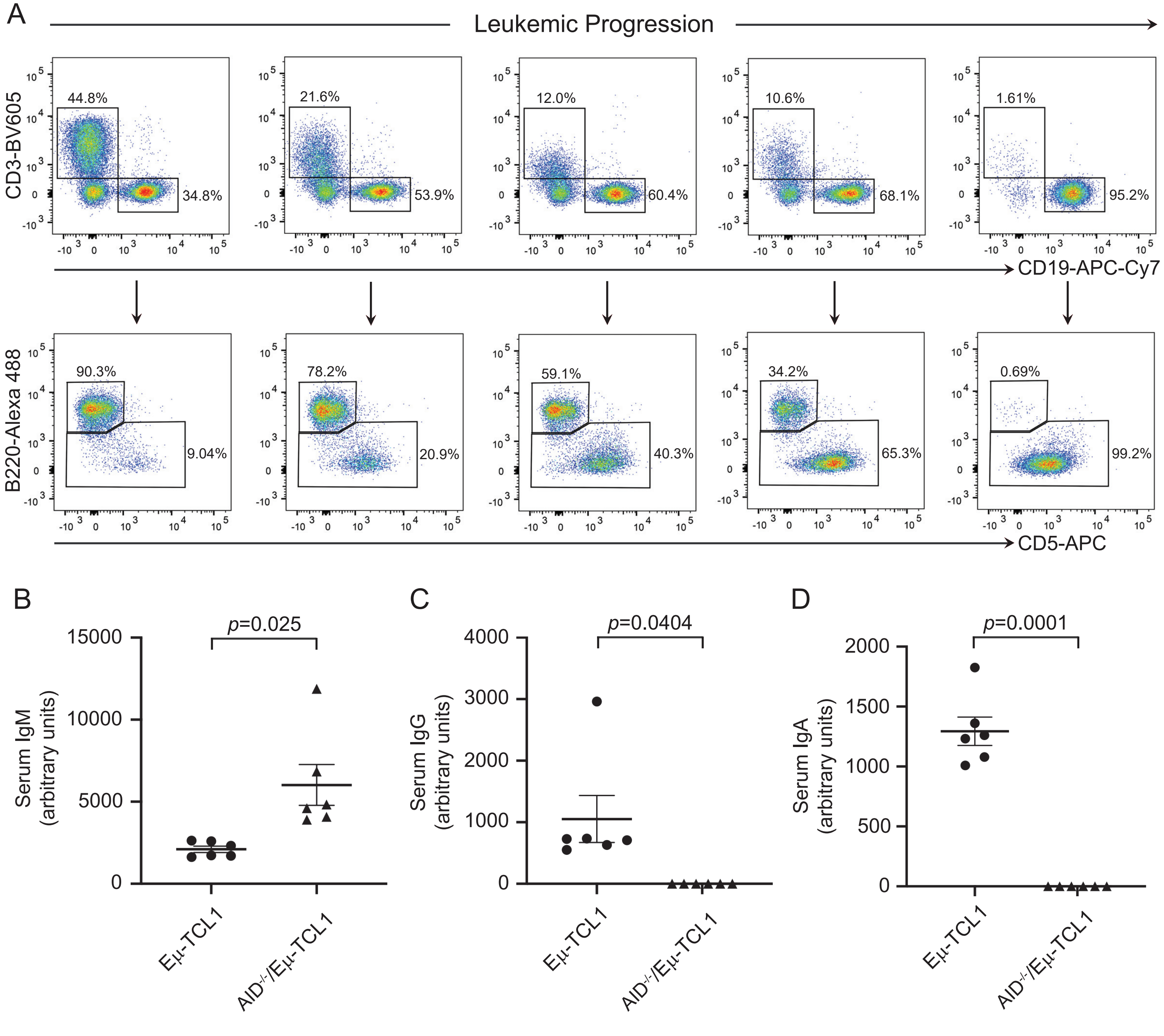
A
Leukemic Progression
CD3-BV605
44.8%
34.8%
21.6%
53.9%
12.0%
60.4%
10.6%
68.1%
1.61%
95.2%
CD19-APC-Cy7
B220-Alexa 488
90.3%
9.04%
78.2%
20.9%
59.1%
40.3%
34.2%
65.3%
0.69%
99.2%
CD5-APC
B
p=0.025
Serum IgM (arbitrary units)
Eμ-TCL1
AID-/-/Eμ-TCL1
C
p=0.0404
Serum IgG (arbitrary units)
D
p=0.0001
Serum IgA (arbitrary units)

Figure S3

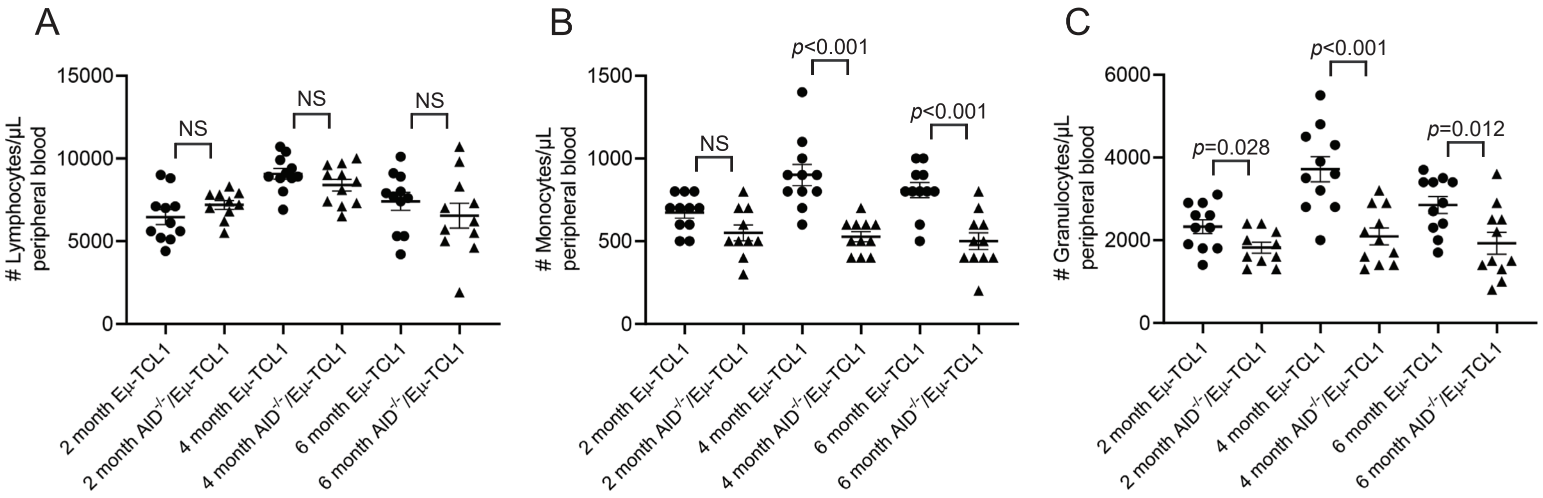
A
Lymphocytes/µL peripheral blood
15000
10000
5000
0
NS
NS
NS
2 month Eµ-TCL1
2 month AID-/-/Eµ-TCL1
4 month Eµ-TCL1
4 month AID-/-/Eµ-TCL1
6 month Eµ-TCL1
6 month AID-/-/Eµ-TCL1
B
Monocytes/µL peripheral blood
1500
1000
500
0
NS
p<0.001
p<0.001
C
Granulocytes/µL peripheral blood
6000
4000
2000
0
p=0.028
p<0.001
p=0.012

Figure S4

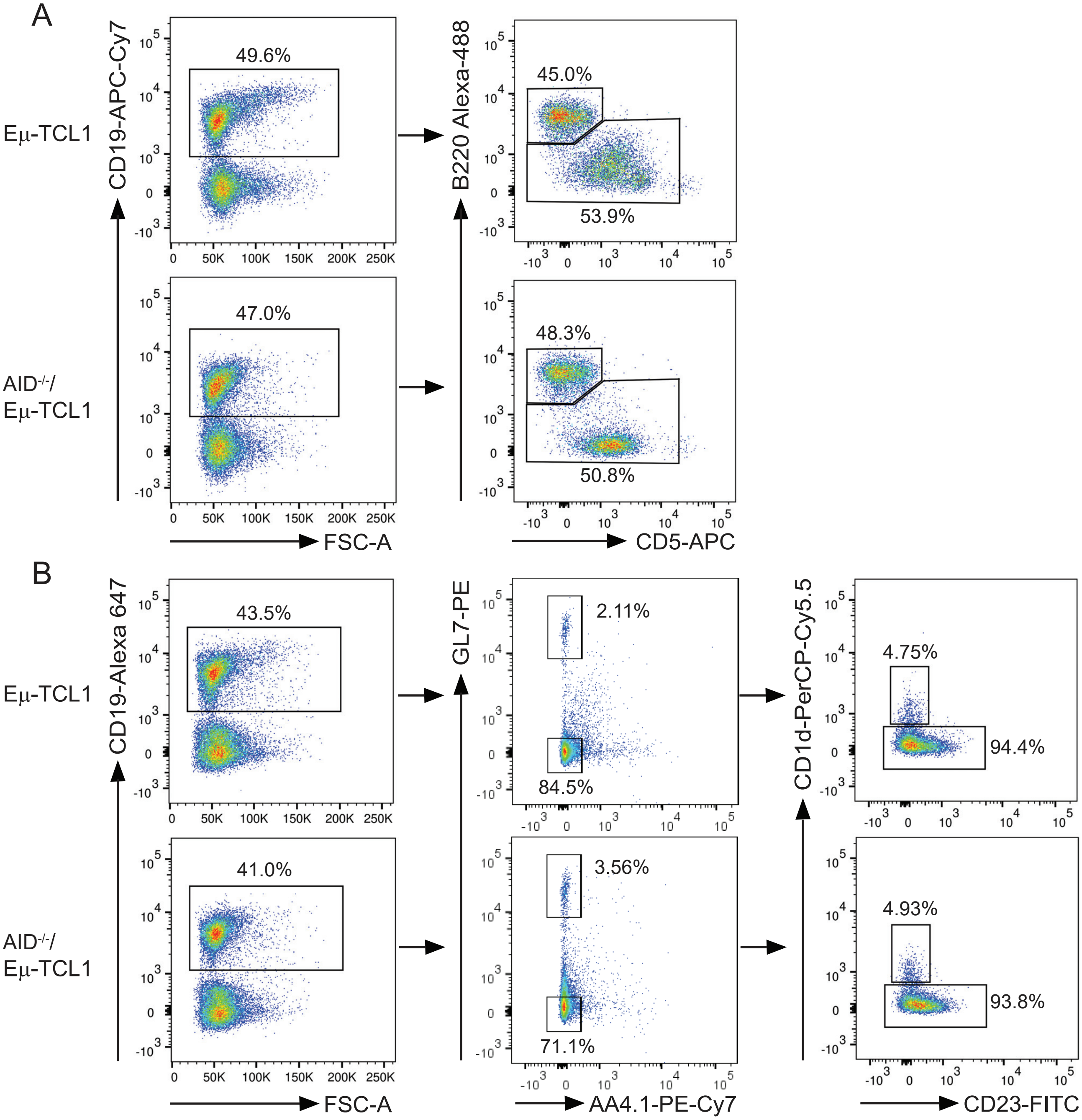
A
Eμ-TCL1
AID-/-/
Eμ-TCL1
CD19-APC-Cy7
49.6%
47.0%
FSC-A
B220 Alexa-488
45.0%
53.9%
48.3%
50.8%
CD5-APC
B
CD19-Alexa 647
43.5%
41.0%
GL7-PE
2.11%
84.5%
3.56%
71.1%
AA4.1-PE-Cy7
CD1d-PerCP-Cy5.5
4.75%
94.4%
4.93%
93.8%
CD23-FITC

Figure S5

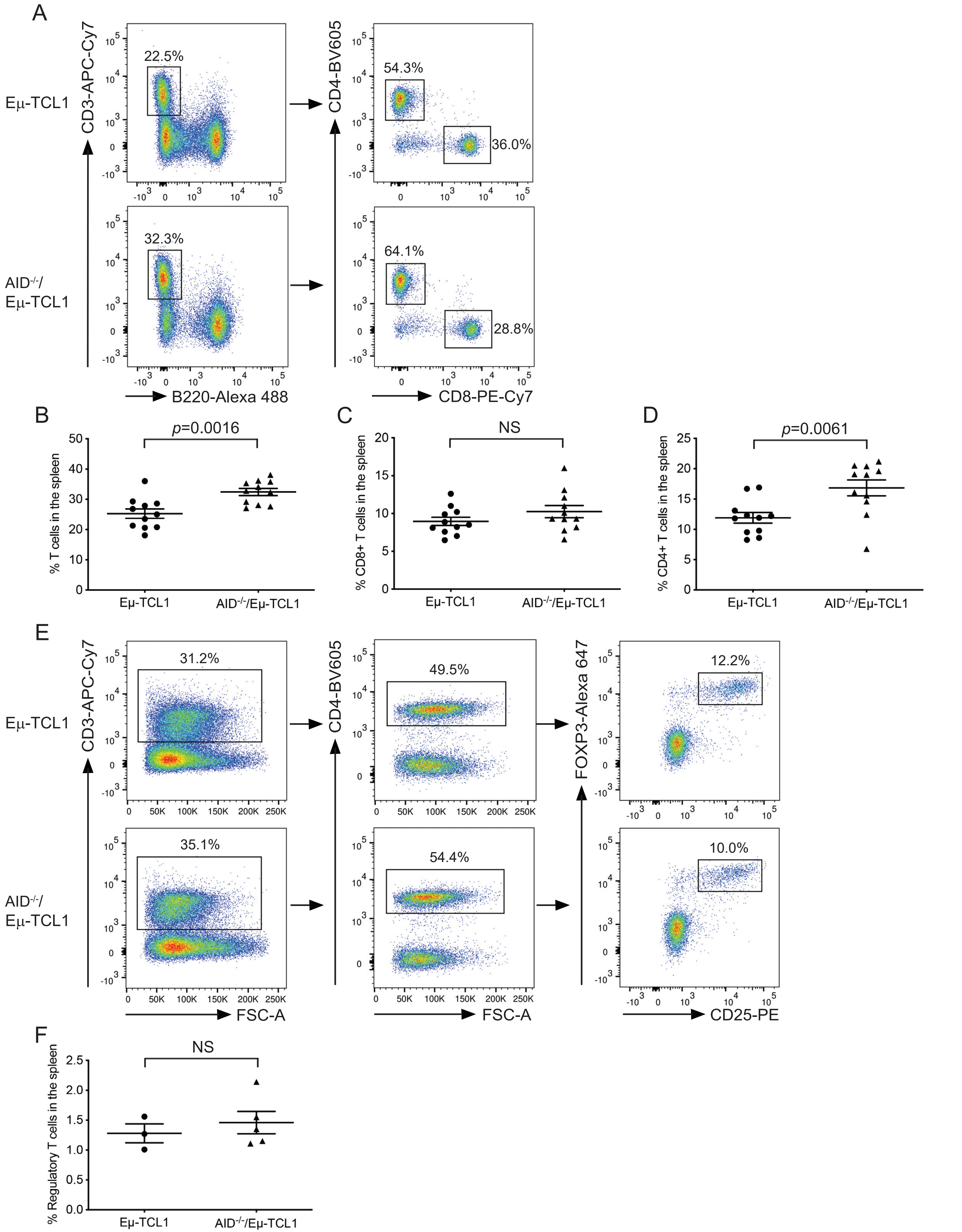
A
Eμ-TCL1
AID-/-/
Eμ-TCL1
CD3-APC-Cy7
B220-Alexa 488
22.5%
32.3%
CD4-BV605
CD8-PE-Cy7
54.3%
36.0%
64.1%
28.8%
B
p=0.0016
% T cells in the spleen
C
NS
% CD8+ T cells in the spleen
D
p=0.0061
% CD4+ T cells in the spleen
Eμ-TCL1
AID-/-/Eμ-TCL1
E
31.2%
35.1%
FSC-A
49.5%
54.4%
FOXP3-Alexa 647
CD25-PE
12.2%
10.0%
F
NS
% Regulatory T cells in the spleen

Figure S6

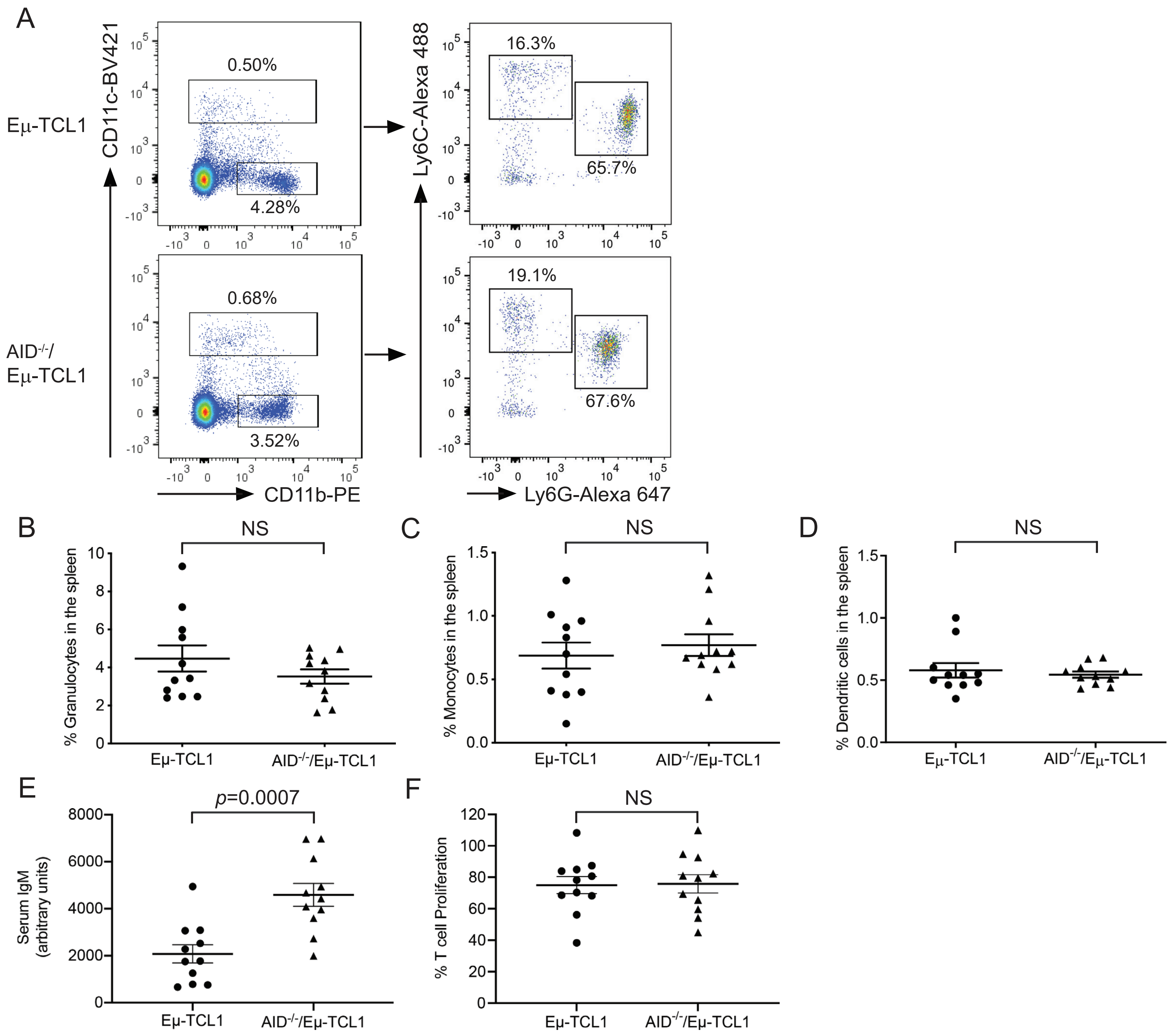
A
Eμ-TCL1
AID-/-/
Eμ-TCL1
CD11c-BV421
CD11b-PE
0.50%
4.28%
0.68%
3.52%
Ly6C-Alexa 488
Ly6G-Alexa 647
16.3%
65.7%
19.1%
67.6%
B
NS
% Granulocytes in the spleen
Eμ-TCL1
AID-/-/Eμ-TCL1
C
NS
% Monocytes in the spleen
D
NS
% Dendritic cells in the spleen
E
p=0.0007
Serum IgM
(arbitrary units)
F
NS
% T cell Proliferation

Figure S7

A

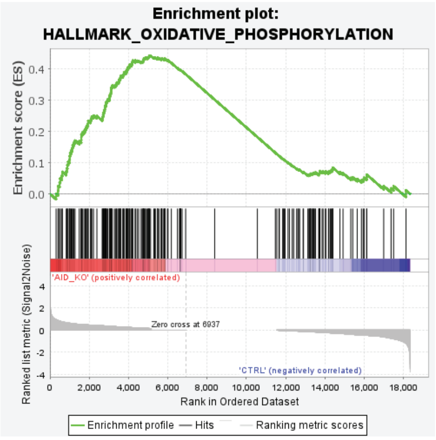

NES = 1.65

B

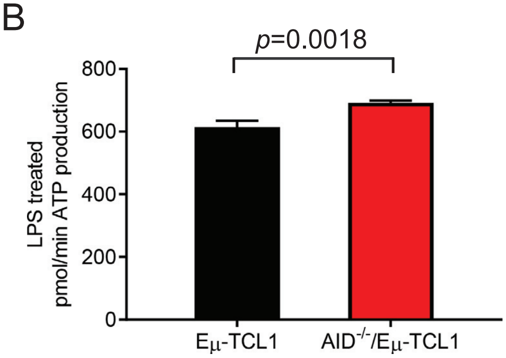

C

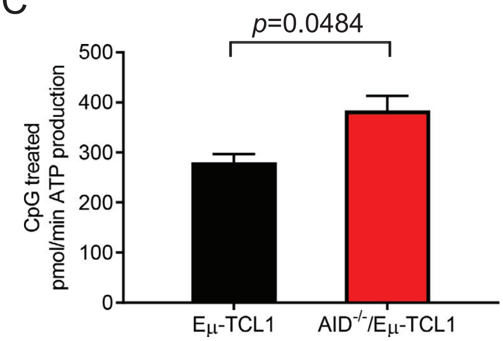

Figure S8

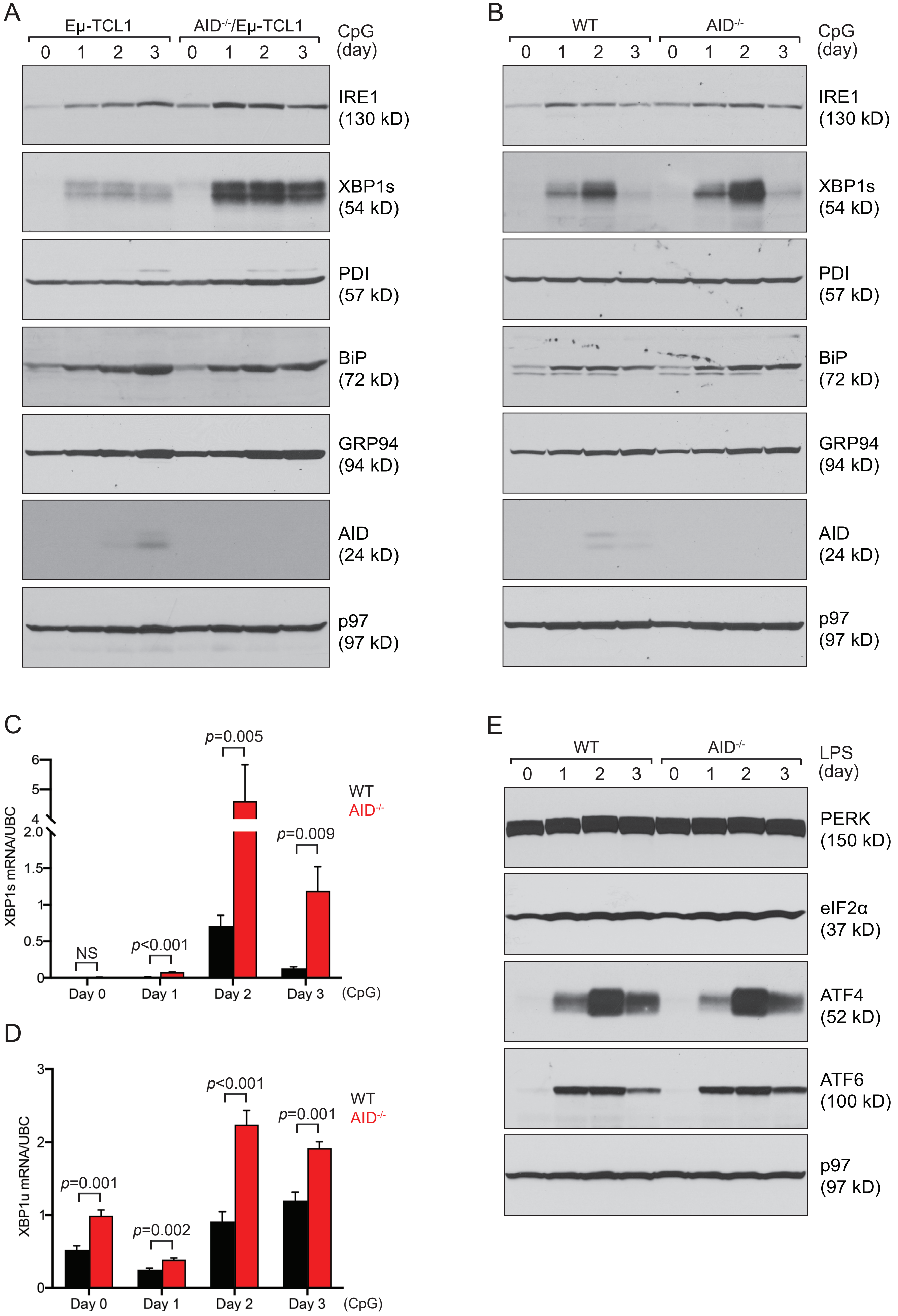
A
Eμ-TCL1
AID-/-/Eμ-TCL1
CpG (day)
0 1 2 3 0 1 2 3
IRE1 (130 kD)
XBP1s (54 kD)
PDI (57 kD)
BiP (72 kD)
GRP94 (94 kD)
AID (24 kD)
p97 (97 kD)
B
WT
AID-/-
CpG (day)
0 1 2 3 0 1 2 3
IRE1 (130 kD)
XBP1s (54 kD)
PDI (57 kD)
BiP (72 kD)
GRP94 (94 kD)
AID (24 kD)
p97 (97 kD)
C
XBP1s mRNA/UBC
WT
AID-/-
NS
p<0.001
p=0.005
p=0.009
Day 0
Day 1
Day 2
Day 3
(CpG)
D
XBP1u mRNA/UBC
WT
AID-/-
p=0.001
p=0.002
p<0.001
p=0.001
Day 0
Day 1
Day 2
Day 3
(CpG)
E
WT
AID-/-
LPS (day)
0 1 2 3 0 1 2 3
PERK (150 kD)
eIF2α (37 kD)
ATF4 (52 kD)
ATF6 (100 kD)
p97 (97 kD)

Figure S9

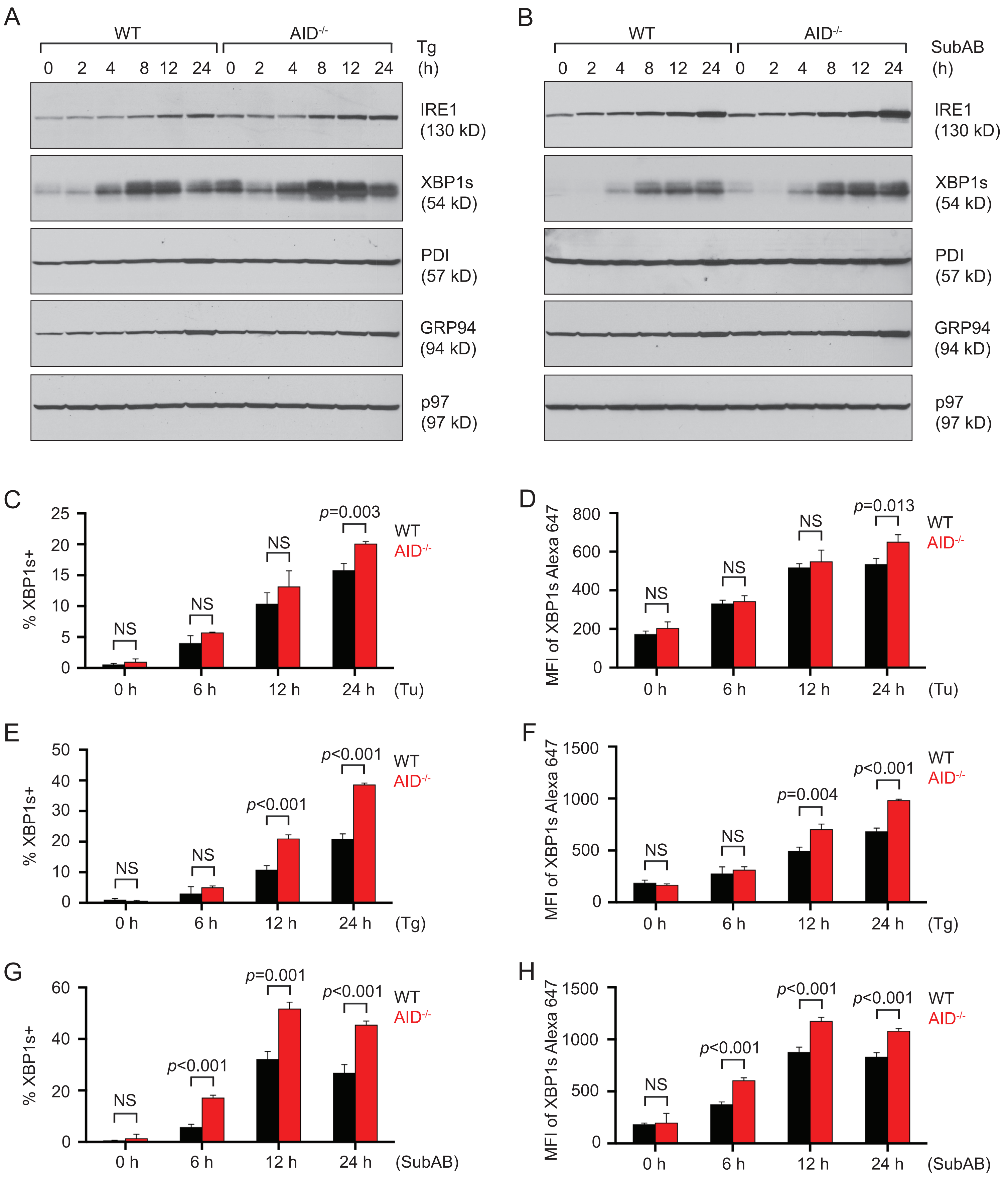
A
WT
AID-/-
Tg
(h)
0 2 4 8 12 24 0 2 4 8 12 24
IRE1 (130 kD)
XBP1s (54 kD)
PDI (57 kD)
GRP94 (94 kD)
p97 (97 kD)
B
WT
AID-/-
SubAB
(h)
0 2 4 8 12 24 0 2 4 8 12 24
IRE1 (130 kD)
XBP1s (54 kD)
PDI (57 kD)
GRP94 (94 kD)
p97 (97 kD)
C
% XBP1s+
0 5 10 15 20 25
0 h 6 h 12 h 24 h (Tu)
NS NS NS p=0.003
WT
AID-/-
D
MFI of XBP1s Alexa 647
0 200 400 600 800
0 h 6 h 12 h 24 h (Tu)
NS NS NS p=0.013
WT
AID-/-
E
% XBP1s+
0 10 20 30 40 50
0 h 6 h 12 h 24 h (Tg)
NS NS p<0.001 p<0.001
WT
AID-/-
F
MFI of XBP1s Alexa 647
0 500 1000 1500
0 h 6 h 12 h 24 h (Tg)
NS NS p=0.004 p<0.001
WT
AID-/-
G
% XBP1s+
0 20 40 60
0 h 6 h 12 h 24 h (SubAB)
NS p<0.001 p=0.001 p<0.001
WT
AID-/-
H
MFI of XBP1s Alexa 647
0 500 1000 1500
0 h 6 h 12 h 24 h (SubAB)
NS p<0.001 p<0.001 p<0.001
WT
AID-/-

Figure S10

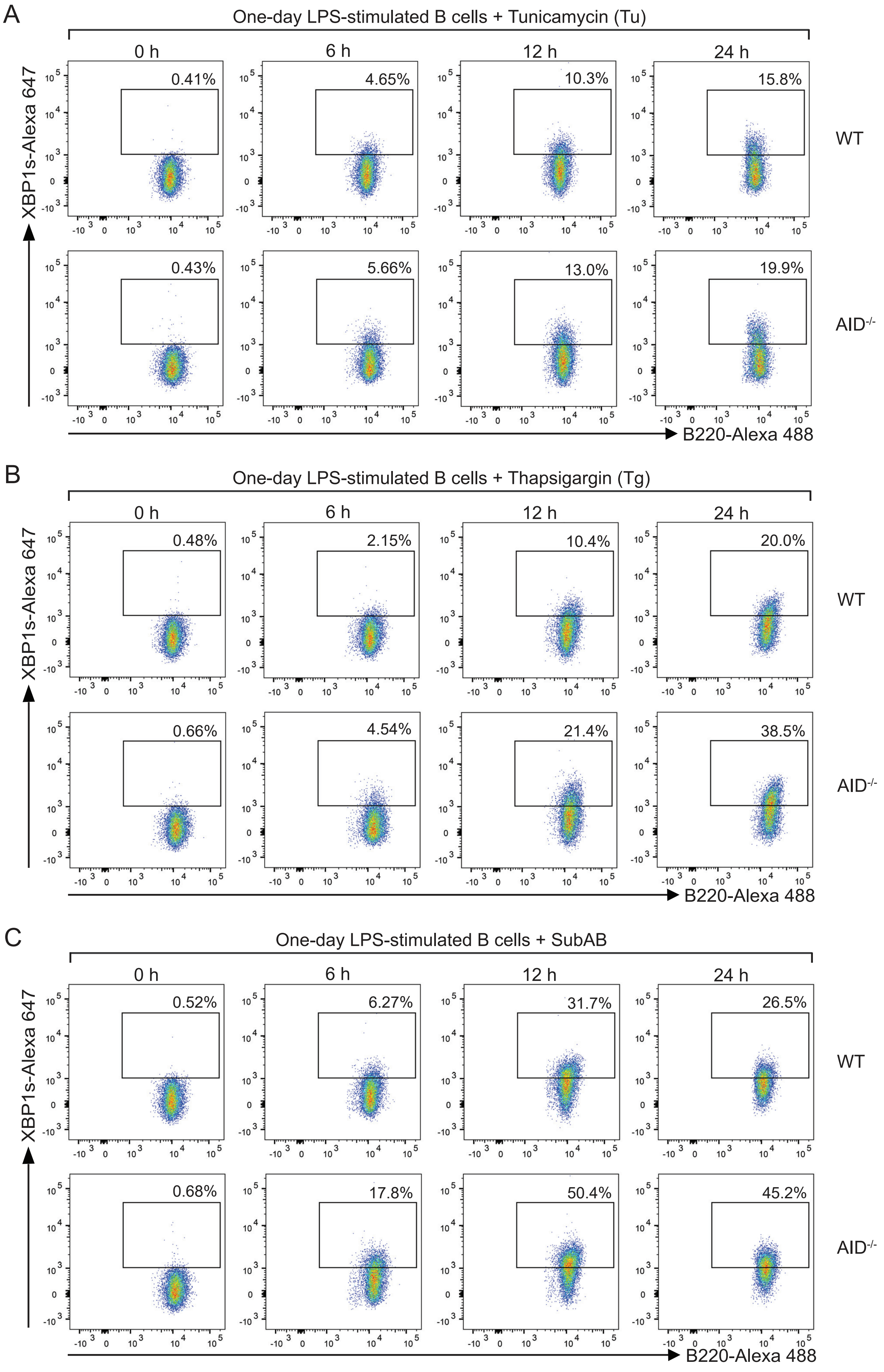
A
One-day LPS-stimulated B cells + Tunicamycin (Tu)
0 h
6 h
12 h
24 h
0.41%
4.65%
10.3%
15.8%
WT
0.43%
5.66%
13.0%
19.9%
AID-/-
XBP1s-Alexa 647
B220-Alexa 488
B
One-day LPS-stimulated B cells + Thapsigargin (Tg)
0 h
6 h
12 h
24 h
0.48%
2.15%
10.4%
20.0%
WT
0.66%
4.54%
21.4%
38.5%
AID-/-
XBP1s-Alexa 647
B220-Alexa 488
C
One-day LPS-stimulated B cells + SubAB
0 h
6 h
12 h
24 h
0.52%
6.27%
31.7%
26.5%
WT
0.68%
17.8%
50.4%
45.2%
AID-/-
XBP1s-Alexa 647
B220-Alexa 488

Figure S11

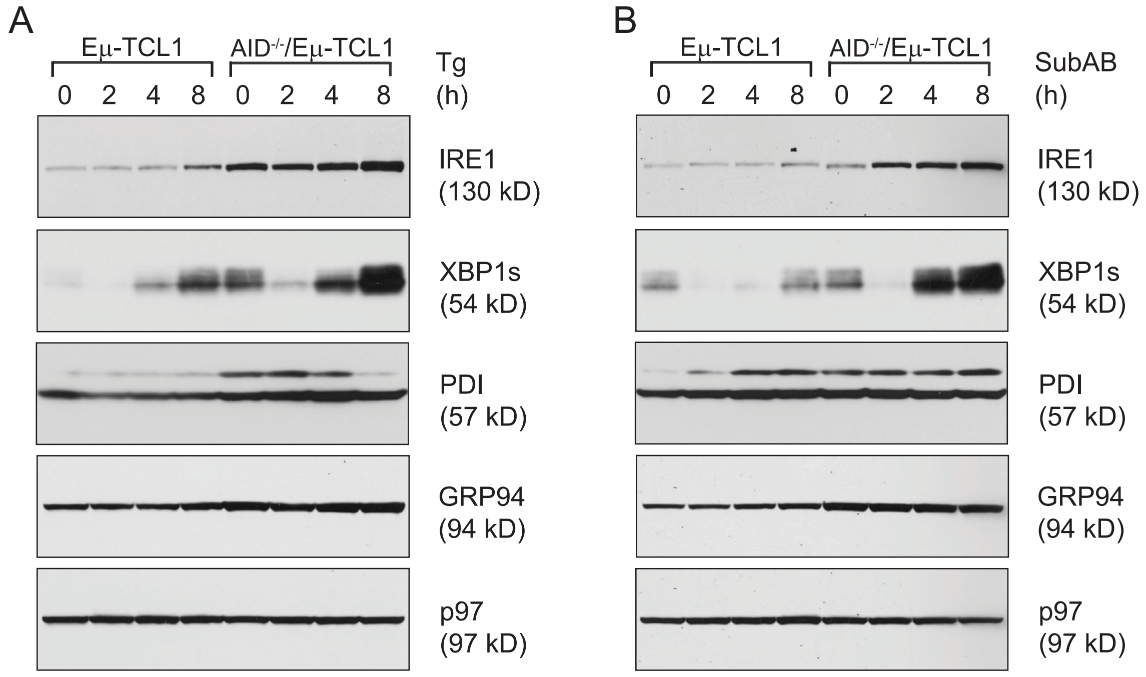

A
Eμ-TCL1
AID-/-/Eμ-TCL1
Tg
(h)
0 2 4 8 0 2 4 8
IRE1
(130 kD)
XBP1s
(54 kD)
PDI
(57 kD)
GRP94
(94 kD)
p97
(97 kD)
B
Eμ-TCL1
AID-/-/Eμ-TCL1
SubAB
(h)
0 2 4 8 0 2 4 8
IRE1
(130 kD)
XBP1s
(54 kD)
PDI
(57 kD)
GRP94
(94 kD)
p97
(97 kD)

Figure S12

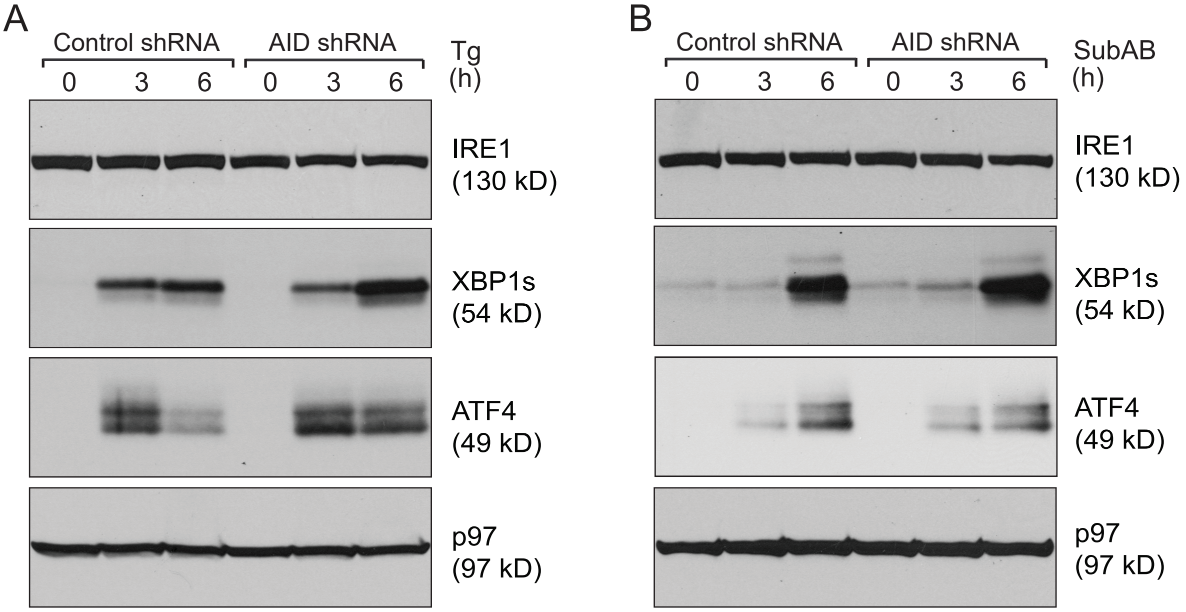

A
Control shRNA
AID shRNA
Tg
(h)
0 3 6 0 3 6
IRE1
(130 kD)
XBP1s
(54 kD)
ATF4
(49 kD)
p97
(97 kD)
B
Control shRNA
AID shRNA
SubAB
(h)
0 3 6 0 3 6
IRE1
(130 kD)
XBP1s
(54 kD)
ATF4
(49 kD)
p97
(97 kD)

Figure S13

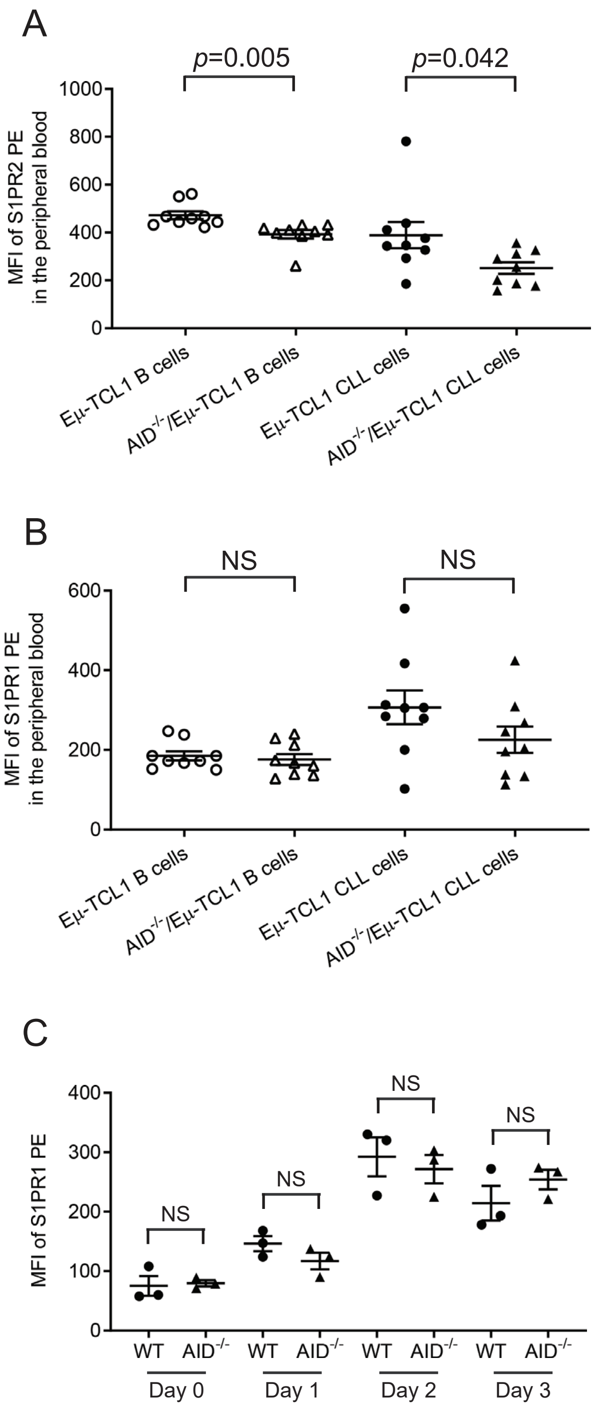
A
p=0.005
p=0.042
MFI of S1PR2 PE in the peripheral blood
1000
800
600
400
200
0
Eμ-TCL1 B cells
AID-/-/Eμ-TCL1 B cells
Eμ-TCL1 CLL cells
AID-/-/Eμ-TCL1 CLL cells
B
NS
NS
MFI of S1PR1 PE in the peripheral blood
600
400
200
0
Eμ-TCL1 B cells
AID-/-/Eμ-TCL1 B cells
Eμ-TCL1 CLL cells
AID-/-/Eμ-TCL1 CLL cells
C
NS
NS
NS
NS
MFI of S1PR1 PE
400
300
200
100
0
WT
AID-/-
Day 0
Day 1
Day 2
Day 3

Figure S14

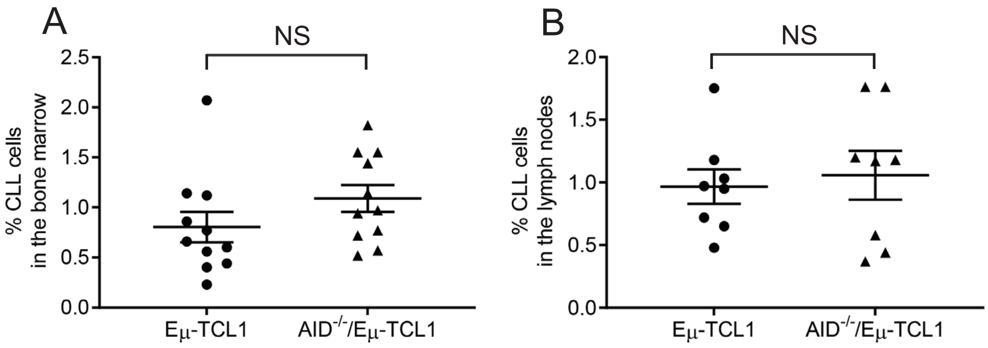

A
NS
% CLL cells
in the bone marrow
2.5
2.0
1.5
1.0
0.5
0.0
Eμ-TCL1
AID-/-/Eμ-TCL1
B
NS
% CLL cells
in the lymph nodes
2.0
1.5
1.0
0.5
0.0
Eμ-TCL1
AID-/-/Eμ-TCL1
